# HSD-Net: a dual-branch CNN-BiLSTM network with hybrid cepstral fusion for heart sound classification

**DOI:** 10.3389/fmedt.2026.1883548

**Published:** 2026-08-18

**Authors:** Caijian Hua, Ye Tian, Liuying Li, Xia Zhou

**Affiliations:** 1 School of Computer Science and Engineering, Sichuan University of Science and Engineering, Yibin, China; 2 Traditional Chinese Medicine Department, Zigong First People’s Hospital, Zigong, China

**Keywords:** cardiovascular diseases screening, CNN-BiLSTM fusion network, heart sound classification, hybrid cepstral fusion, squeeze-and-excitation attention

## Abstract

**Introduction:**

Cardiovascular diseases (CVDs) represent a major global health threat, making early detection crucial. While cardiac auscultation is cost-effective, its reliance on clinical expertise leads to significant variability in diagnostic accuracy. Existing automated heart sound classification methods suffer from inadequate feature representation and limited model performance due to constraints imposed by the randomness, nonstationarity, and complex acoustic environment of phonocardiogram (PCG) signals.

**Methods:**

In this paper, an improved dual-branch CNN-BiLSTM network with hybrid cepstral fusion, named HSD-Net, is proposed for automated heart sound classification. To overcome feature representation limitations, we introduce a hybrid cepstral fusion feature set that combines Mel-Frequency Cepstral Coefficients (MFCC) and Inverse Mel-Frequency Cepstral Coefficients (IMFCC) with their first-order delta coefficients, capturing complementary low-frequency and high-frequency acoustic information. This feature set is processed by a dedicated dual-branch architecture: a convolutional neural network (CNN) branch extracts localized spectrotemporal patterns, while a bidirectional Long Short-Term Memory (BiLSTM) branch models long-range temporal dependencies. A Squeeze-and-Excitation (SE) attention mechanism is integrated to adaptively recalibrate feature importance.

**Results:**

Extensive evaluations on two public datasets demonstrate that the proposed HSD-Net achieves superior performance compared with existing methods. On the PhysioNet/Computing in Cardiology Challenge 2016 dataset, the model achieved an accuracy of 96.25%, a sensitivity of 95.85%, and a specificity of 96.50%. On the additional five-class Yaseen dataset, the model achieved an average accuracy of 99.32% in valvular heart disease classification. Ablation studies quantitatively confirmed that both the hybrid features and the dual-branch architecture contribute significantly to the overall performance.

**Discussion:**

The proposed HSD-Net provides an effective framework for automated heart sound analysis. This work offers a reliable technical foundation for developing clinical decision-support systems, with significant potential for application in early screening and telemedicine.

## 1 Introduction

### 1.1 Research background

**Figure 1:**
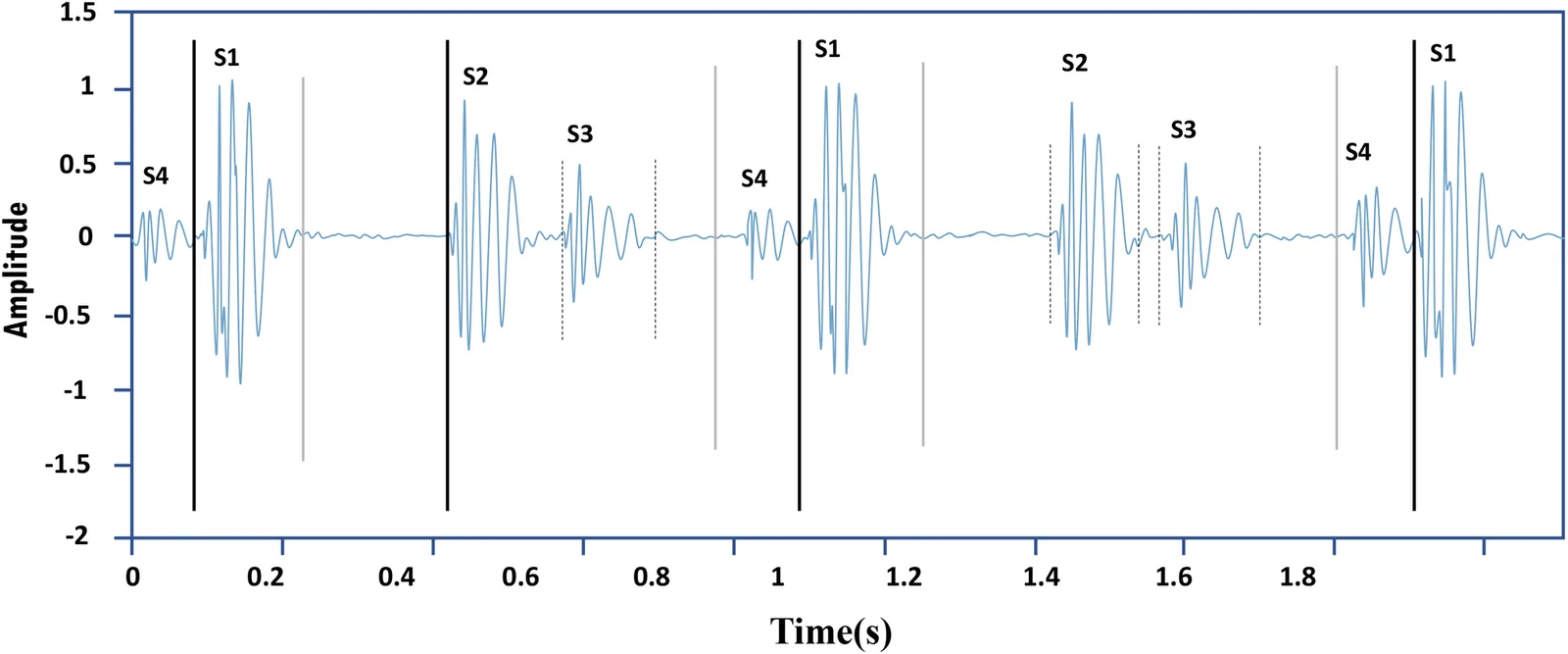
Waveform illustration of heart sounds S1–S4 during systole and diastole.

Cardiovascular diseases (CVDs) impose a substantial and continuously increasing global health burden across both economically developed regions and settings with limited medical resources. The World Health Organization (WHO) continues to identify CVDs as the leading cause of death worldwide, accounting for approximately one-third of all global deaths (1). This burden has increased steadily over recent decades, with CVD-related deaths rising from 12.1 million in 1990 to 18.6 million in 2019 (2). Beyond mortality, CVDs are also associated with long-term disability, recurrent hospitalization, reduced quality of life, and considerable socioeconomic costs. Many cardiovascular abnormalities, particularly valvular lesions and early-stage functional disorders, may remain clinically silent or present with non-specific symptoms before progressing to severe complications. Therefore, timely screening and early risk identification are essential for improving prognosis, reducing delayed diagnosis, and alleviating the burden on healthcare systems, especially in primary care, community, and resource-limited settings. Heart sounds arise from cardiac mechanical activity during the cardiac cycle and provide important acoustic information about cardiovascular function. The first heart sound (S1) indicates the onset of ventricular systole and is mainly associated with atrioventricular valve closure, whereas the second heart sound (S2) marks the onset of ventricular diastole and is related to semilunar valve closure. Additional heart sounds, such as S3 and S4, may also carry clinical significance and are commonly associated with rapid ventricular filling and atrial contraction against a stiff ventricle, respectively. Moreover, valvular abnormalities often produce murmurs due to turbulent blood flow, and changes in heart sound timing, intensity, morphology, and spectral content may reflect stenosis, regurgitation, or other hemodynamic abnormalities. As a digital recording of cardiac acoustic activity, the PCG provides a noninvasive and low-cost signal source for preserving, visualizing, and analyzing these clinically meaningful patterns. Figure 1 shows representative waveforms of S1 through S4 within a single cardiac cycle.

Cardiac auscultation remains a frontline tool in cardiovascular assessment because it is rapid, noninvasive, inexpensive, and convenient for routine screening. However, conventional auscultation is inherently subjective and highly dependent on the clinician’s experience, auditory sensitivity, and surrounding acoustic environment. Its diagnostic reliability can be affected by background noise, patient variability, recording location, and the subtle or transient nature of certain pathological murmurs. Previous studies have reported that diagnostic accuracy can reach approximately 80% for expert cardiologists (3), whereas primary care physicians without dedicated auscultation training typically achieve only 20%–40% accuracy (4). This performance gap highlights the need for objective, reproducible, and accessible automated heart sound analysis, which may help reduce dependence on individual experience and support cardiovascular screening in primary care, community settings, and settings with limited medical resources.

### 1.2 Related work

Computer-aided analysis of heart sounds typically follows a standard processing pipeline comprising three core stages: (1) signal preprocessing, (2) feature extraction, and (3) classifier design. Within this framework, the quality of the extracted features and the architecture of the classifier are crucial, as they largely determine the overall system performance. Consequently, considerable research effort over the past decades has focused on developing more discriminative feature representations and constructing high-performance classifiers. Early mainstream approaches in this field relied on traditional machine learning models. For example, Springer et al. (5) employed a hidden semi-Markov model (HSMM) combined with four envelope features for heart sound segmentation and achieved an average F1 score of 95% using a logistic regression classifier. Building on this probabilistic framework, Oliveira et al. (6) proposed an HSMM with an adaptive dwell time mechanism. Their approach incorporated a Gaussian dwell-time distribution, whose parameters were iteratively optimized using the expectation-maximization (EM) algorithm, while the hidden state sequence was decoded with the Viterbi algorithm. Mei et al. (7) proposed a heart sound classification method that integrates signal quality assessment with the wavelet scattering transform: wavelet scattering coefficients unfolded along the scale dimension are used as input features to a support vector machine (SVM), and the final decision is obtained via scale wise voting. On the PhysioNet/CinC Challenge 2016 dataset, the method achieved 92.23% accuracy, 96.62% sensitivity, 90.65% specificity, and a mean accuracy (MAcc) of 93.64%. Fuadah et al. (8) developed an MFCC-based machine learning pipeline for heart sound classification. Nia and Hesar (9) presented an abnormal heart sound detection framework that segments PCG recordings into four cardiac-cycle components (S1, systole, S2, and diastole) and constructs a 116-dimensional feature vector composed of power spectral density (PSD)-based time-frequency descriptors and time-domain statistical features. On the PhysioNet/CinC Challenge 2016 dataset, by combining the synthetic minority over-sampling technique (SMOTE) with 10-fold cross-validation, their best configuration sequential forward floating selection (SFFS) with a radial basis function kernel support vector machine (RBF-SVM) achieved 98.03% accuracy, 97.64% sensitivity, and 98.43% specificity. Karan and Padhi (10) proposed a feature-engineering approach for PCG-based heart valve disease recognition by mapping wavelet packet decomposition into the Hilbert domain to extract packet instantaneous frequency deviation (PIFD) and packet instantaneous energy deviation (PIED) features. Using an ECOC-based multiclass scheme with SVM/KNN base learners, they reported unweighted average recall (UAR) of 99.80% and 99.32% on a GitHub five-class dataset and a Firat University ten-class dataset, respectively. In addition, Vimalajeewa et al. (11) investigated murmur detection using a wavelet-domain multiscale “scale/complexity” feature set combined with lightweight classifiers; with class-weighted learning, the SVM achieved 76.61% accuracy (82.12% sensitivity and 54.02% specificity) and an area under the receiver operating characteristic curve (AUC) of 0.741 on the CirCor DigiScope dataset.

Over the past few years, the rapid advancement of artificial intelligence has made deep learning models, including CNN models, recurrent neural network (RNN) models, and architectures using attention mechanisms, highly effective research tools for heart sound classification. Related studies have mainly evolved along two directions: (1) end-to-end representation learning directly from raw PCG signals, and (2) hybrid pipelines that couple explicit feature construction with deep classifiers. Along the end-to-end classification line, Deng et al. (12) proposed a deep learning framework for distinguishing normal and abnormal heart sounds. In this framework, an enhanced MFCC-based feature representation is extracted from PCG recordings and then fed into a convolutional recurrent neural network (CRNN) for classification. Their approach avoids explicit cardiac-cycle segmentation; instead, MFCC features are extracted directly from the recordings, and dynamic coefficients are further incorporated to characterize short-term variations between adjacent frames. The resulting MFCC sequences are then fed into a hybrid architecture, achieving strong classification performance on the PhysioNet/CinC 2016 dataset. Das and Dandapat (13) introduced a multi-kernel residual convolutional network (MK-RCNN). Their method encodes PCG segments through parallel, multi-scale residual convolutional blocks, and then concatenates the branch-wise outputs into a unified multi-kernel representation to capture discriminative local patterns at different scales. In addition, to mitigate training bias induced by class imbalance, they adopt a class-weighted (cost-sensitive) loss during optimization. Importantly, the study goes beyond reporting classification accuracy by also examining model size and computational complexity, offering a more deployment-oriented assessment of practical feasibility. In parallel, Latif et al. (14) used segmented PCG sequences and MFCC features to compare gated recurrent architectures, including LSTM, GRU, BiLSTM, and BiGRU, for temporal modeling in heart sound classification. The results showed that bidirectional recurrent modeling, especially BiLSTM, was more stable and had stronger discriminative power than the other models. Zhang et al. (15) proposed an integrated computer-aided diagnosis (CAD) system designed for on-device deployment, using log-Mel spectrograms as input and combining a dual-branch convolutional architecture with channel/spatial attention (CSA) to emphasize key time-frequency information. However, the experiments were conducted on an in-house dataset with a relatively small sample size and limited data diversity from a single or narrowly sourced collection. Along the hybrid processing direction, Li et al. (16) extracted a 497-dimensional feature set and fed it into a tailored CNN for binary classification. The network uses stacked convolution and pooling modules, with global average pooling at the output. Meintjes et al. (17) transformed signals into time-frequency representations via continuous wavelet transform (CWT) and used a convolutional neural network to classify S1 and S2 heart sounds, achieving an accuracy of 86.0%. Zhang et al. (18) fused Mel-cepstral coefficients with second-order spectral features and reorganized the combined descriptors into a feature image for CNN-based learning. The CNN architecture was redesigned with reference to AOCT- and VGG-style backbones to better exploit the complementary information carried by cepstral and higher-order spectral representations. Experiments on a five-class dataset of 1,000 recordings with a 3:2 train/test split achieved 99.6% training accuracy and 98.5% test accuracy. Al-Tam et al. (19) benchmarked four deep models for PCG-based diagnosis, including VGG16, ResNet50, a Transformer encoder, and a hybrid ResNet50–Transformer–MLP framework. Their pipeline uses MFCC features as input, with z-score normalization, discrete cosine transform (DCT), and data augmentation strategies. Under five-fold cross-validation, the hybrid model achieved strong performance on both the Yaseen dataset and the PhysioNet/CinC Challenge 2016 dataset, demonstrating its effectiveness across different evaluation benchmarks. Li et al. (20) proposed CAFusionNet, which fuses multi-scale features from different depths within a residual backbone and uses a lightweight channel-attention module (GCT) to reweight feature responses, enhancing discriminative information while keeping parameter overhead low. Xiong et al. (21) proposed a two-stage framework where SAM-BiLSTM segments PCG recordings to infer cardiac-cycle states, and SE-MFCNN extracts and integrates state-specific features for classification. SE-based attention enhances the fusion module, improving classification performance. Experimental results show that combining segmentation priors with attention-enhanced CNN classification effectively boosts heart sound diagnostic performance. Beyond these software-based digital learning frameworks, optical information processing has also been investigated as a complementary direction for efficient heart sound recognition. One representative study Mohapatra et al. (22) proposed a hybrid joint transform correlator (JTC)-based optical recognition framework, in which heart sound signals were first converted into spectrograms and then recognized through optical correlation. To account for intra-class variability in heart sound spectrograms, the method further incorporated a composite filter synthesized from multiple spectrograms within each class into the JTC framework. The feasibility of this optical correlator-based approach was validated through both numerical simulations and optical experiments, suggesting its potential as a high-speed alternative to conventional digital recognition pipelines.

Although the aforementioned studies have achieved substantial progress in automated heart sound classification, there remains room for improvement. (1) At the feature representation level, current heart sound classification is challenged by the complex and highly variable cardiac acoustic environment. In practical recording scenarios, phonocardiogram (PCG) recordings often have a low signal to noise ratio (SNR) and may be affected by ambient noise and electromyographic interference. Differences in recording devices and acquisition conditions can further introduce distribution shifts, reducing the coverage and stability of pathological cues. One line of work uses MFCC as the main input for sequential modeling or hybrid learning frameworks (14, 19), while another relies on a single time-frequency transform (17). In addition, some studies adopt large, multi-domain handcrafted feature banks (16), or combine cepstral descriptors with higher-order spectral features (18). In this context, although preprocessing such as bandpass filtering can partially suppress certain interferences, residual noise and distribution discrepancies across scenarios remain unavoidable; consequently, if a representation is biased toward particular frequency bands or provides only a “static spectral envelope” without explicitly characterizing temporal evolution between frames, discriminative cues are more likely to be lost under acquisition changes or distribution shifts. Time-frequency transforms like CWT provide multi-scale information, but their effectiveness depends on scale and resolution choices, making them sensitive to noise and parameter settings. In scenarios with low SNR, they may introduce redundant structures and blur key patterns. Large handcrafted feature sets can capture diverse aspects of PCG signals but rely heavily on feature engineering and may be sensitive to device variability and acquisition changes; high dimensional descriptors also risk overfitting, especially in noisy and imbalanced data. To address these challenges, we propose a hybrid cepstral fusion representation based on “frequency band complementarity” and “dynamic enhancement.” MFCC is used for low-frequency components, while IMFCC shifts focus to higher frequencies, enhancing sensitivity to faint high-frequency cues and improving modeling under noisy conditions. Additionally, first-order delta features, ΔMFCC and ΔIMFCC, capture temporal evolution across frames, complementing static cepstral coefficients and addressing the lack of dynamic modeling in traditional features. (2) At the model design level, most deep learning approaches for heart sound classification are built primarily on CNN or RNN models and have further evolved into multi-scale, attention-enhanced, and hybrid variants. However, in real-world screening scenarios, these models often struggle to reconcile local details with cycle-level temporal structure. Specifically, one line of work focuses on CNN models and strengthens local discriminative pattern extraction through multiscale convolutions and attention mechanisms, as exemplified by Li et al. (20) and Zhang et al. (15). Such methods that rely mainly on CNN models are generally effective at capturing localized spectrotemporal textures and salient frequency patterns, yet they often rely on implicit hierarchical stacking to absorb long-range dependencies, which makes it difficult to explicitly model temporal evolution across complete cardiac cycles. In contrast, another line of work adopts RNN-based architectures for explicit temporal modeling. For example, Latif et al. (14) systematically compared LSTM, GRU, BiLSTM, and BiGRU using MFCC sequences extracted from segmented PCG recordings, and reported that bidirectional recurrent modeling—particularly BiLSTM—tends to be more stable and discriminative. Taken together, architectures that emphasize either local texture learning alone or global temporal modeling alone often fall short of capturing both types of critical information required for effective heart sound diagnosis. Motivated by this, we propose a parallel CNN-BiLSTM dual-branch fusion framework, and introduce SE channel attention in both the convolutional branch and the fusion between branches stage to enable adaptive reweighting, thereby better exploiting complementary cues in the learned representations.

Based on the above issues, we propose HSD-Net, a dual-branch CNN-BiLSTM network with hybrid cepstral fusion for heart sound classification. The main contributions of this work are summarized as follows:

1. A hybrid cepstral fusion feature set is developed by integrating MFCC, IMFCC, and their first-order delta coefficients to encode complementary low-frequency and high-frequency acoustic information and short-term spectral dynamics in a unified feature space.
2. A dual-branch CNN-BiLSTM fusion network is constructed to capture both localized spectrotemporal patterns and long-range cardiac-cycle dependencies, with squeeze-and-excitation attention to enhance discriminative feature fusion.
3. Comprehensive experiments and ablation studies on the PhysioNet/CinC Challenge 2016 dataset benchmark and the Yaseen five-class dataset demonstrate the effectiveness of HSD-Net.

The rest of the paper is organized as follows. Section 2 describes the data and methods, including preprocessing, feature extraction, and the design of the HSD-Net architecture. Section 3 presents the experimental setup and result analysis. Section 4 discusses the main findings and key design choices, and summarizes the strengths and limitations of the proposed method. Finally, Section 5 concludes the paper and outlines future work.

## 2 Materials and methods

**Figure 2:**
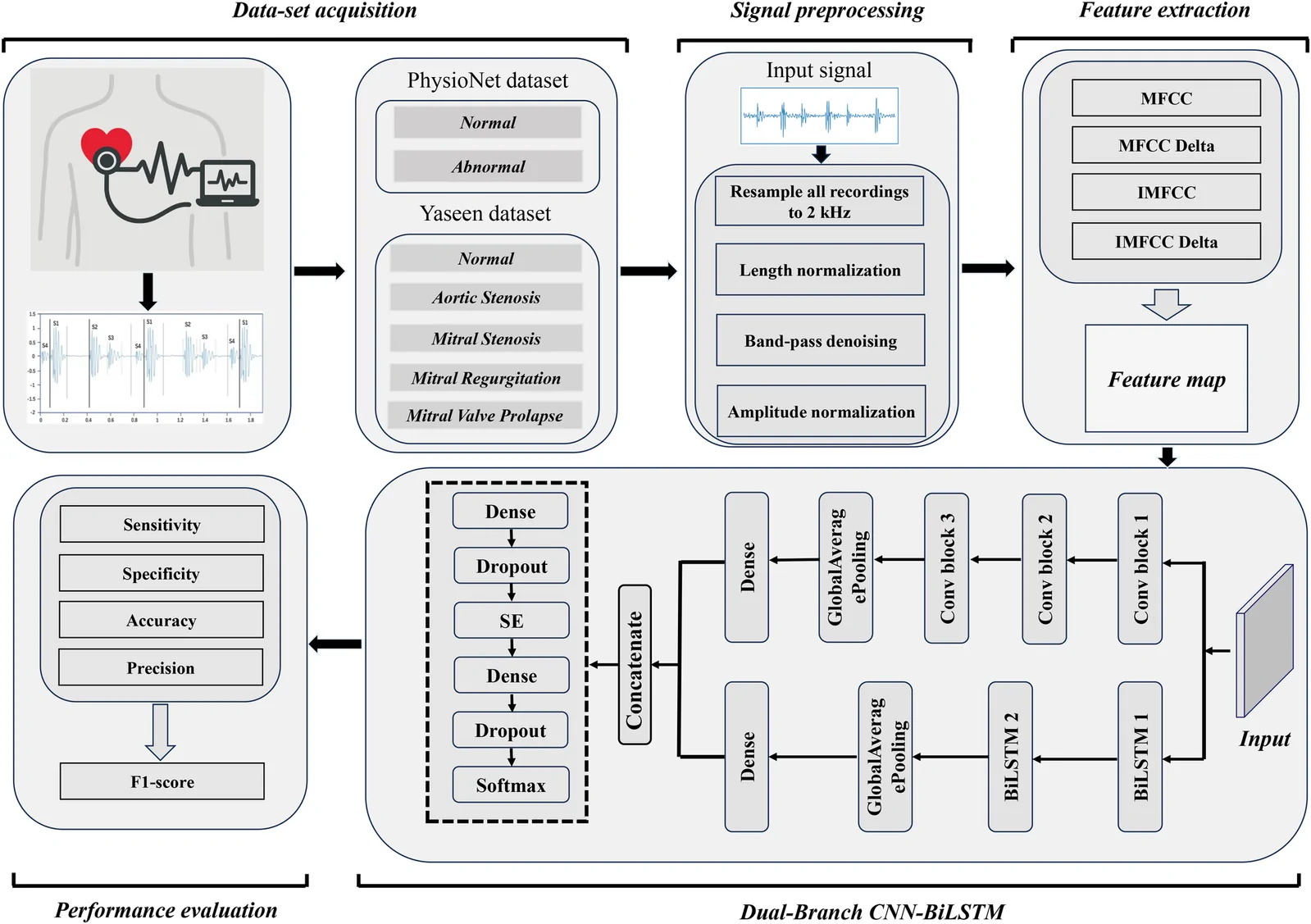
Overall framework of the proposed HSD-Net.

The overall framework of the proposed HSD-Net can be summarized into the following steps: (1) data acquisition; (2) signal preprocessing; (3) feature extraction; (4) classification with performance evaluation. In the data acquisition stage, two public heart sound datasets were adopted, including the PhysioNet/CinC Challenge 2016 dataset benchmark and the Yaseen five class heart disease dataset. During preprocessing, all recordings were resampled to 2 kHz, with duration normalization, bandpass filtering for denoising, and amplitude normalization applied to ensure consistent signal quality and length. The standard MFCC and IMFCC features, together with their first-order delta coefficients (ΔMFCC and ΔIMFCC), were extracted from the preprocessed heart sound signals to form the model input feature set. In the final stage, these features were used as input to the CNN-BiLSTM model for classification and performance evaluation. The overall framework of HSD-Net is illustrated in Figure 2.

### 2.1 Data description and preprocessing

The primary dataset selected in this study was obtained from the 2016 PhysioNet/Computing in Cardiology (CinC) Challenge database (23) and included heart sound recordings collected from multiple clinical and non-clinical resources. This database is organized into six training subsets (a–f), containing a total of 3,240 heart sound recordings from 1,297 subjects, including both healthy individuals and patients with cardiovascular diseases such as valvular defects and coronary artery disease. These recordings were acquired in a variety of clinical and non-clinical environments using different models of electronic stethoscopes. All recordings were annotated by clinical experts as either normal or abnormal, resulting in 665 normal recordings from healthy subjects and 2,575 abnormal recordings from patients with cardiac disease. The duration of the recordings ranges from 5 s to 120 s, and all signals were resampled to 2,000 Hz. To standardize the input length while preserving sufficient cardiac-cycle context, the recordings were segmented into fixed 4 s clips, and each generated clip inherited the label of its source recording. To avoid potential clip-level data leakage, cross-validation partitioning was performed at the original recording level before segmentation, so that clips generated from the same recording were assigned to the same subset. Recordings or remaining segments shorter than 4 s were excluded during data preparation and were not used for subsequent feature extraction, model training, or evaluation. To address class imbalance during training, class weights were computed from the class distribution of the training subset within each cross-validation iteration and incorporated into the loss function during model optimization. The validation subset and the held-out test subset were not used for class-weight estimation. The details of the PhysioNet/CinC Challenge 2016 dataset are shown in Table 1.

**Table 1:** PhysioNet/computing in cardiology (CinC) challenge 2016 dataset.

File name	Environment	Normal	Abnormal
Training-a	Home/environment	292	117
Training-b	Hospital	104	386
Training-c	Hospital	24	7
Training-d	Hospital/laboratory	28	27
Training-e	Hospital/laboratory	183	1,958
Training-f	Hospital	34	80
Total	–	665	2,575

For further analysis, a secondary dataset was also tested in this study, which included heart sound recordings obtained from the Yaseen dataset released on GitHub by Yaseen et al. (24). The dataset comprised a total of 1,000 phonocardiogram recordings from subjects with normal (N) heart sounds and four types of valvular heart disease (AS, MS, MR, and MVP), with 200 recordings in each category. These recordings were compiled from multiple public databases and clinical studies, and only signals with relatively good quality and low noise levels were retained. Each recording was provided as a mono-channel signal sampled at 8,000 Hz with an effective duration of 2 s, a window length sufficient to cover at least one complete cardiac cycle under typical resting heart-rate conditions. In this study, all recordings were resampled to 2,000 Hz to match the primary dataset signals for subsequent feature extraction and classification. The details of the Yaseen dataset are shown in Table 2.

**Table 2:** Yaseen dataset.

File name	Heart sound condition	Number of recordings
N	Normal	200
AS	Aortic stenosis	200
MS	Mitral stenosis	200
MR	Mitral regurgitation	200
MVP	Mitral valve prolapse	200

**Figure 3:**
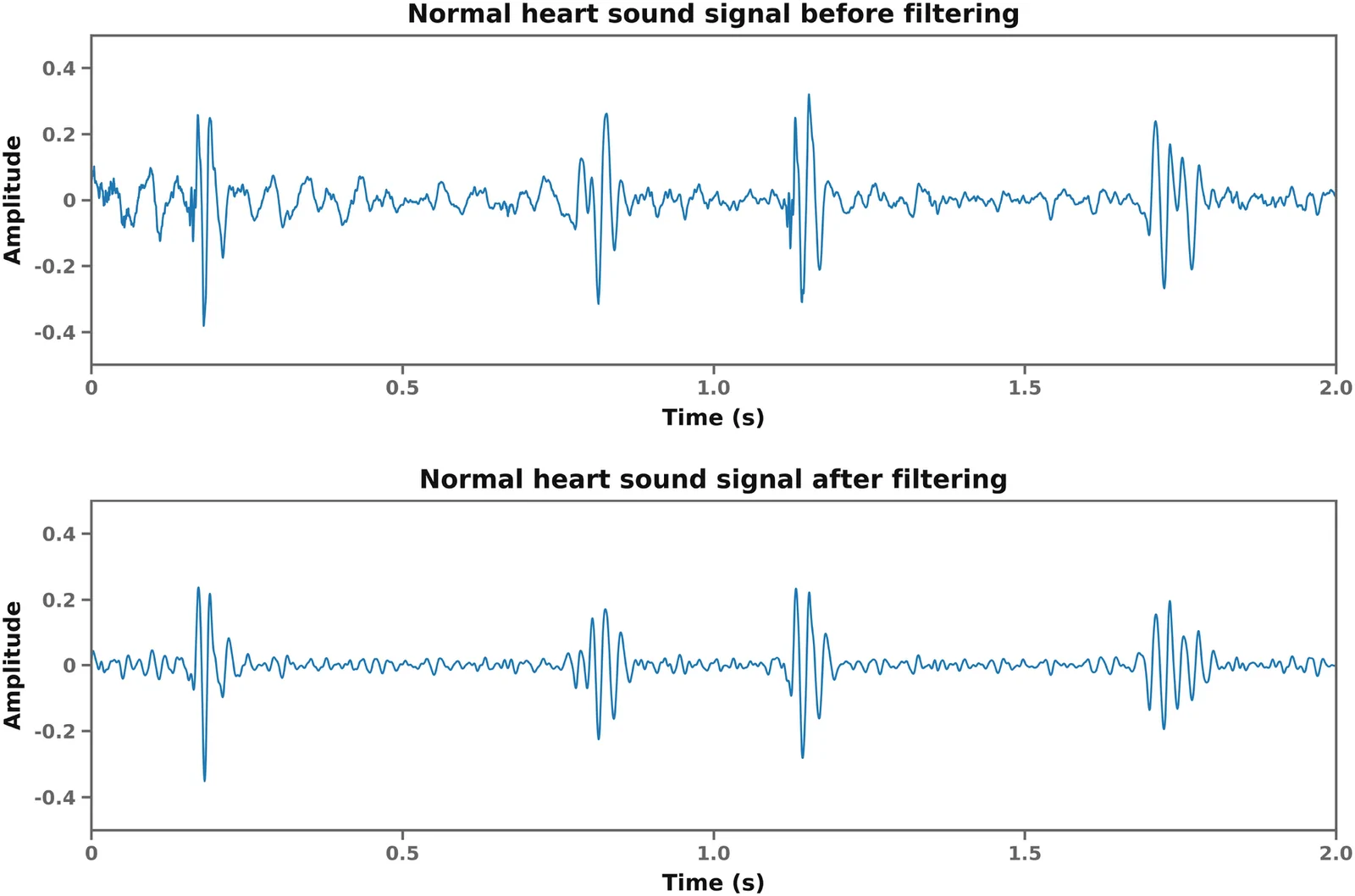
Original and filtered signals of normal heart sounds.

**Figure 4:**
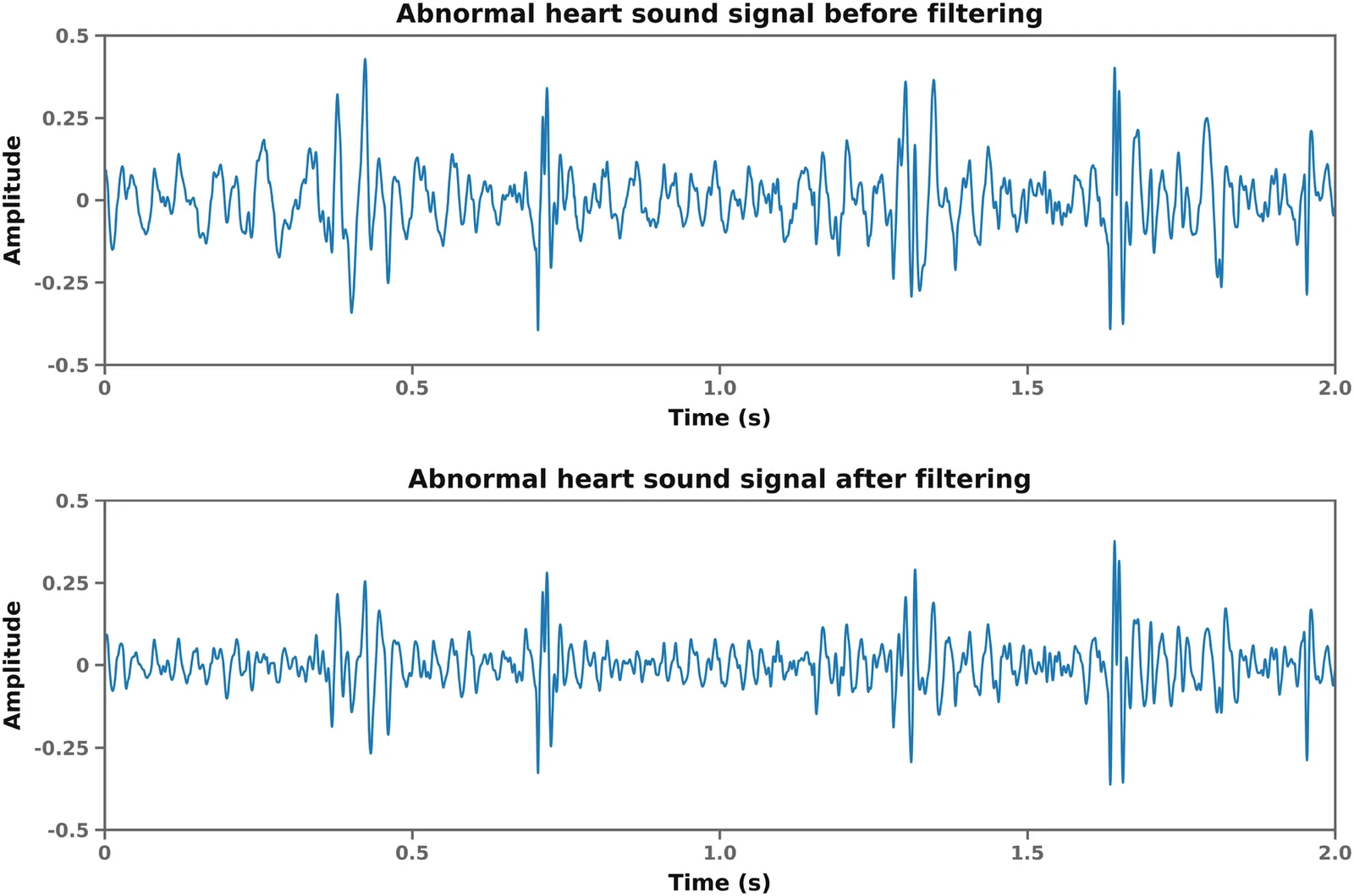
Original and filtered signals of abnormal heart sounds.

To ensure consistent signal quality and comparability across the two datasets, a unified heart sound preprocessing pipeline was adopted. First, a fifth-order Butterworth bandpass filter was applied to the raw PCG recordings. Considering that certain pathological murmurs may exhibit spectral energy beyond 400 Hz, and that IMFCC is introduced in this study to better characterize subtle high-frequency components, using an overly narrow upper cutoff may attenuate clinically relevant information. Therefore, the passband was set to 20–600 Hz, aiming to suppress environmental noise and electromyographic interference while retaining potentially informative higher-frequency murmur content. Compared with commonly used settings such as 20–200 Hz or 20–400 Hz, the 20–600 Hz configuration provides broader coverage of high-frequency murmur-related components and is more consistent with the feature design of this work. In addition, we further examined wider passband settings of 20–700 Hz and 20–800 Hz under the same experimental protocol to assess the impact of preserving even higher-frequency components. The filtered recordings were then uniformly resampled to 2,000 Hz. For duration normalization, the PhysioNet/CinC Challenge 2016 recordings were segmented into fixed-length 4 s clips, whereas the Yaseen recordings retained their original 2 s duration. Recordings or remaining segments shorter than the required input duration were excluded from analysis. Figures 3, 4 illustrate the heart sound signals before and after filtering, respectively.

### 2.2 Hybrid cepstral features for heart sound representation

#### 2.2.1 MFCC-based heart sound representation

As the first component of the hybrid cepstral feature extraction stage in HSD-Net, MFCC-based heart sound representation relies on a Mel-scale frequency analysis of the PCG signals. By mapping the signals onto the Mel scale, which approximates human auditory perception, the filterbank emphasizes the spectral envelope and highlights low-frequency components associated with primary heart sounds. Given the similarities between heart sounds and speech signals in both their generation mechanisms and temporal nonstationarity, MFCC is highly suitable for cardiac signal analysis. Previous studies have shown that MFCC can effectively characterize physiological and pathological patterns in phonocardiograms and achieve high recognition accuracy in heart sound classification tasks (25). Therefore, MFCC was selected as a foundational feature in this study. The procedure for MFCC feature extraction can be described as follows:

1. Pre-emphasis
  A pre-emphasis operation is applied to the heart sound signal to amplify relatively weak high-frequency components and suppress random noise. The pre-emphasis high-pass filter is denoted as H(z), and its mathematical expression is given in Equation (1):
  $$H(z)=1-\beta z^{-1}$$ (1)
  Here, β denotes the gain coefficient of the filter, typically ranging between 0 and 1, and $z^{-1}$ represents the delay factor, indicating the time delay of the signal.
2. Framing
  To facilitate subsequent analysis, the pre-emphasized heart sound signal is segmented into short, quasi-stationary frames using an overlapping window. This overlap ensures smooth transitions between adjacent frames. The total number of frames is denoted by $N_{f}$ and calculated using Equation (2):
  $$N_{f}=\left\lfloor \frac{L-w_{l}}{c}\right\rfloor +1,$$ (2)
  where L is the length of the discrete-time heart sound signal, $w_{l}$ is the frame length, c is the frame shift between two consecutive frames, and ⌊⋅⌋ denotes the floor operator that returns the greatest integer less than or equal to its argument. In this study, all recordings from both datasets were resampled to 2,000 Hz before feature extraction. The frame length $w_{l}$ was set to 100 samples, corresponding to 50 ms, and the frame shift c was set to 40 samples, corresponding to 20 ms. Consequently, the signal length L was 8,000 samples for the 4 s segments from the PhysioNet/CinC Challenge 2016 dataset and 4,000 samples for the 2 s recordings from the Yaseen dataset.
3. Windowing
  In the preprocessing stage, a Hamming window is applied to the heart sound signals to mitigate spectral leakage, thereby improving the accuracy of subsequent analyses. The Hamming window function W(n) is defined in Equation (3):
  $$W(n)=(1-\gamma )-\gamma \cos\;\left(\frac{2\pi n}{w_{l}-1}\right),\;0\leq n\leq w_{l}-1.$$ (3)
  Here, W(n) denotes the Hamming window coefficient at the n-th sample within a frame, n is the discrete-time sample index, γ is a constant empirically set to 0.46 (26).
4. Fast fourier transform
  Through this procedure, the energy distribution within the signal’s frequency spectrum can be derived, and the corresponding formula is given in Equation (4):
  $$X(i,k)=F\left[x_{i}(n)\right]$$ (4)
  Here, X(i,k) denotes the k-th frequency component of the i-th frame in the frequency domain after the Fourier transform, F[⋅] denotes the fast Fourier transform (FFT), $x_{i}(n)$ represents the time-domain heart sound signal of the i-th frame, i is the frame index, n is the time-domain sample index, and k indexes the FFT frequency bin.
5. Mel filter
  A Mel filterbank, composed of a series of triangular filters, is applied to the spectrum obtained from the Fast Fourier Transform (FFT). The transfer function for each filter is defined in Equation (5):
  $$H_{m}(l)=\left\{ \begin{array}{cc} 0, & l<f(m-1)\\ \frac{l-f(m-1)}{f(m)-f(m-1)}, & f(m-1)\leq l\leq f(m)\\ \frac{\;f(m+1)-l}{f(m+1)-f(m)}, & f(m)<l\leq f(m+1)\\ 0, & l>f(m+1) \end{array}\right.$$ (5)
  Here, $H_{m}(l)$ denotes the response of the m-th Mel filter at the l-th frequency bin, l and m are the indices of the frequency bin and Mel filter, respectively. f(⋅) maps a Mel filter index to the corresponding frequency, and M is the total number of Mel filters in the filterbank.
6. Calculating energy values
  The filtered spectral energy is obtained by computing the dot product between the signal’s energy spectrum and each filter’s frequency response within the Mel filter bank. This operation is formulated in Equation (6):
  $$S(i,m)=\sum_{k=0}^{w_{l}-1}E(i,k)H_{m}(k),\;0\leq m<M$$ (6)
  Here, S(i,m) denotes the energy output of the m-th Mel filter for the i-th frame, and E(i,k) is the energy spectrum of the i-th frame at the k-th frequency bin; $H_{m}(k)$ denotes the frequency response of the m-th Mel filter at the k-th frequency bin, k indexes the FFT frequency bin, and M denotes the number of Mel filters in the filterbank, which is set to 40 in this study.
7. Calculating discrete cosine transform (DCT) cepstrum
  The cepstral coefficients are obtained by first applying a logarithm to the Mel-filtered energies and then performing a Discrete Cosine Transform (DCT). This is expressed in Equation (7):
  $$mfcc(i,p)=\sqrt{\frac{2}{M}}\sum_{m=0}^{M-1}\log\;\left[S(i,m)\right]\cos\left(\frac{\pi p(2m-1)}{2M}\right)$$ (7)
  where mfcc(i,p) denotes the p-th MFCC coefficient of the i-th frame, and p denotes the cepstral order for the 13 retained coefficients.

#### 2.2.2 IMFCC-based heart sound representation

**Figure 5:**
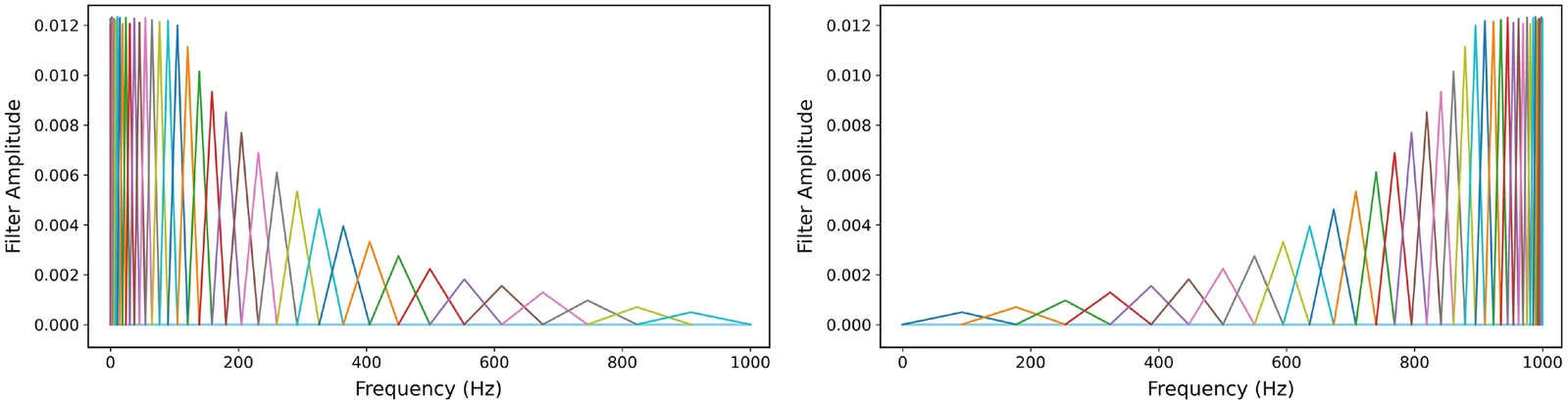
Mel filter bank (left) and inverted Mel filter bank (right).

As a complementary variant of MFCC, IMFCC enhances sensitivity to high-frequency information by inverting the distribution of the traditional Mel filter bank. In the conventional MFCC framework, filters are concentrated in the low-frequency region, whereas IMFCC shifts the emphasis toward the high-frequency spectrum (Figure 5). This high-frequency focus is particularly important for capturing subtle yet informative pathological events, such as murmurs and valvular defects, which may be insufficiently represented by standard MFCC features. By incorporating this complementary spectral information, IMFCC provides a more comprehensive characterization of cardiac acoustic signals.

Initially, a 40-filter bank was applied to derive MFCC and IMFCC feature representations. After the discrete cosine transform, the first 13 cepstral coefficients were retained for each feature type. This setting provides a compact representation with reduced redundancy and is commonly used in PCG based classification tasks.

#### 2.2.3 Dynamic feature extraction

A fundamental limitation of static spectrogram coefficients is that they capture only the spectral envelope within a single frame and thus fail to represent inter-frame temporal dynamics. To address this and better reflect the dynamic nature of cardiac acoustics, we incorporate first-order delta coefficients, which capture rapid spectral and energy variations associated with S1/S2 heart sounds and pathological murmurs, thereby enhancing the discriminative power of the feature set. For both MFCC and IMFCC, the corresponding delta vectors are truncated to the first 13 coefficients, consistent with the processing of the static features. The first-order delta coefficients (ΔMFCC and ΔIMFCC) are computed using Equation (8):

$$d(i)=\frac{1}{\sqrt{\sum_{t=-r}^{r}t^{2}}}\sum_{t=-r}^{r}t\;C(i+t)$$ (8)

where d(i) denotes the first-order delta coefficient at frame i, C(i+t) is the MFCC or IMFCC parameter at frame i+t, t is the regression offset index, and r is the regression window size, which is set to 2 in this study.

#### 2.2.4 Hybrid cepstral fusion

Although MFCC effectively captures low-frequency components, relying on it alone may lead to the loss of critical high-frequency information. To overcome this limitation and exploit the complementary high-frequency sensitivity of IMFCC, this study adopts a hybrid cepstral fusion strategy that combines MFCC-based and IMFCC-based feature representations for heart sound classification. The fused feature is defined in Equation (9) as

$$M_{\text{\textbackslash{},fusion}}=\left(M_{\text{MFCC}},\;\Delta M_{\text{MFCC}},\;M_{\text{IMFCC}},\;\Delta M_{\text{IMFCC}}\right)$$ (9)

where $M_{\text{\textbackslash{},fusion}}$ denotes the hybrid cepstral fusion feature; $M_{\text{MFCC}}$ and $\Delta M_{\text{MFCC}}$ denote the static MFCC features and their first-order deltas; $M_{\text{IMFCC}}$ and $\Delta M_{\text{IMFCC}}$ denote the static IMFCC features and their first-order deltas.

**Figure 6:**
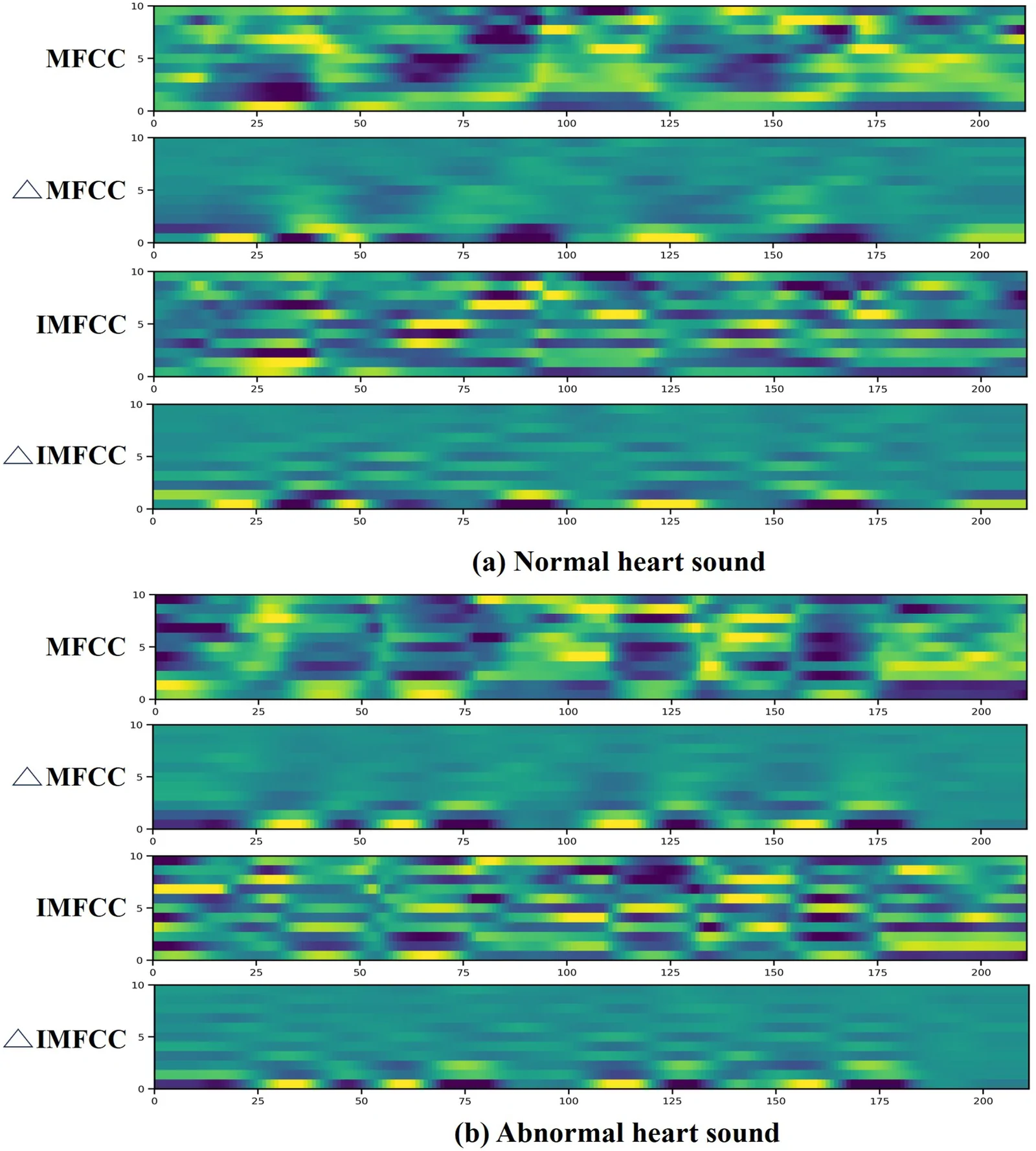
2D visualization of the cepstral features: (**a**) normal heart sound; (**b**) abnormal heart sound.

**Figure 7:**
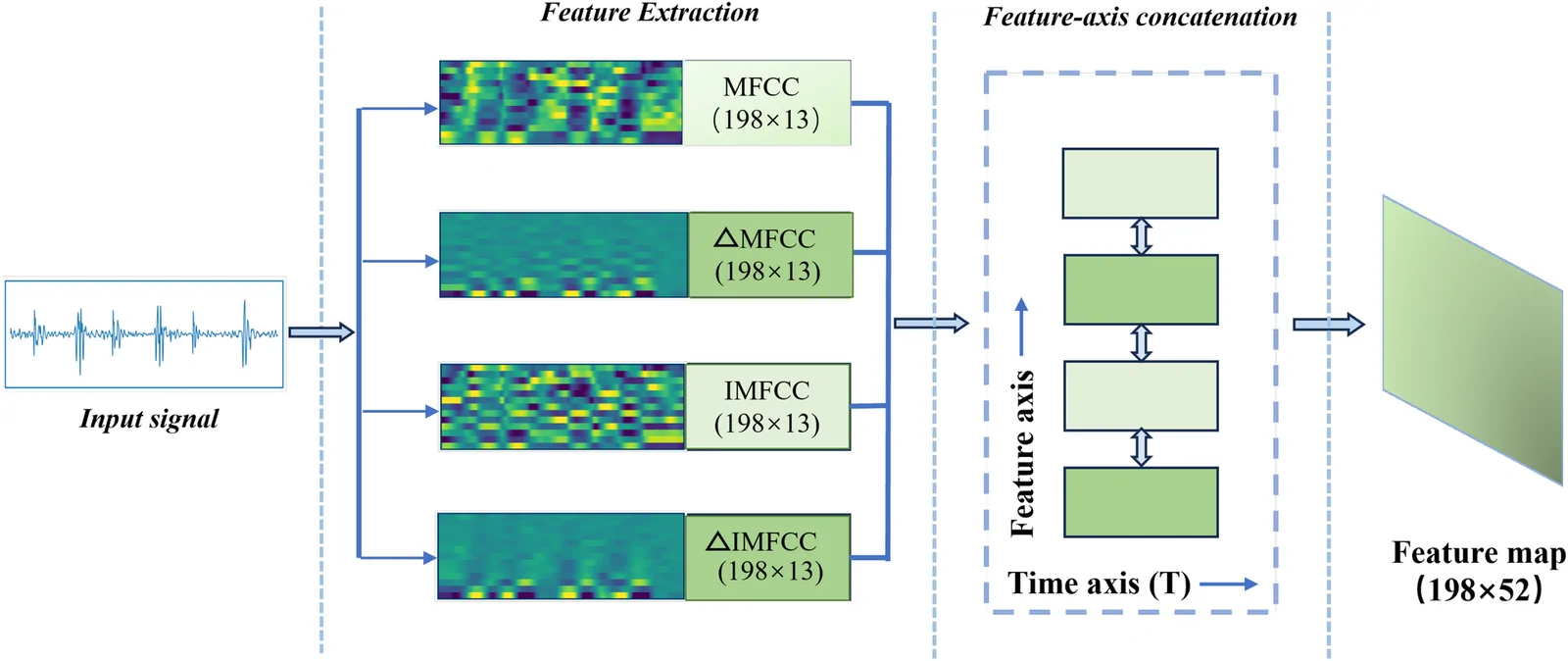
Hybrid cepstral feature fusion.

Figure 6 shows 2D visualizations of four feature types for normal and abnormal heart sounds. From top to bottom, the panels are MFCC, ΔMFCC, IMFCC, and ΔIMFCC, each with a size of 198×13. As illustrated in Figure 7, these four feature matrices are concatenated along the feature dimension to form a 198×52 frame-wise cepstral feature matrix, where 198 denotes the number of time frames and 52 denotes the feature dimension of each frame. This matrix is then used as the neural network input.

### 2.3 Architecture of the proposed HSD-Net

**Figure 8:**
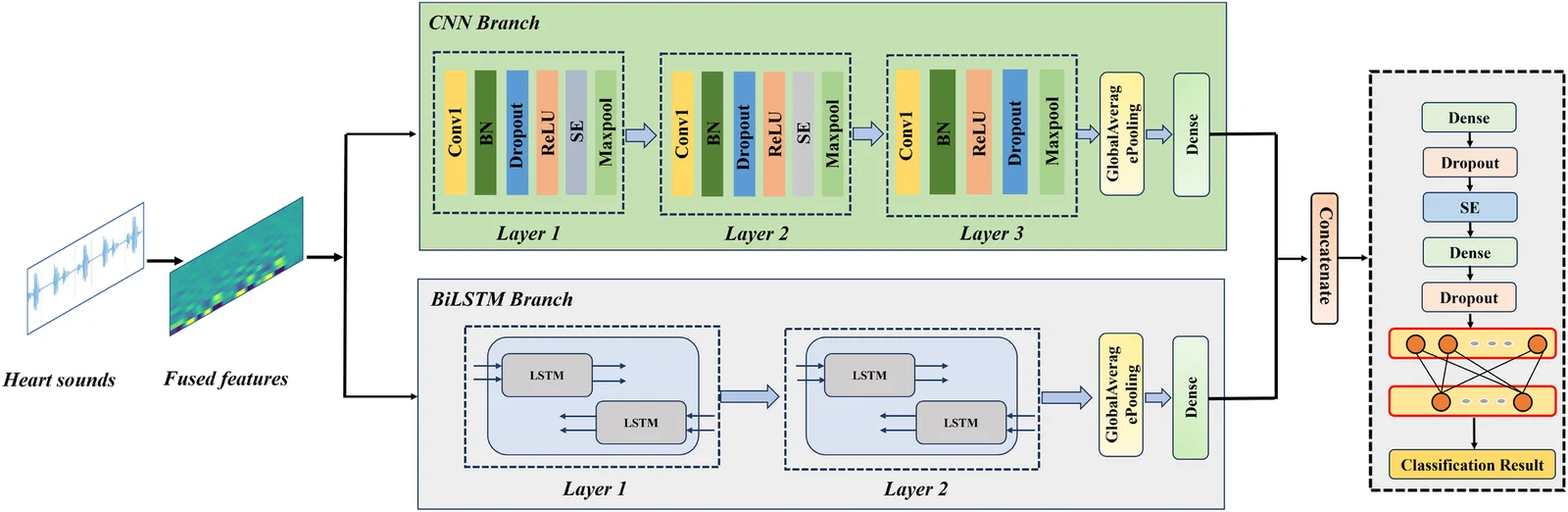
The overall network structure of the proposed HSD-Net.

The proposed HSD-Net employs a dual-branch architecture that integrates a convolutional neural network (CNN) with a long short-term memory (LSTM) network. This design enables the joint modeling of both the spectral–spatial structure and the temporal dynamics of input heart sound signals. The architecture is inspired by Zheng et al. (27), which reported that ensemble-style models generally achieve superior performance. To further enhance feature representation, a squeeze-and-excitation (SE) attention mechanism is embedded in both the convolutional branch and the fusion layer. By adaptively recalibrating channel-wise feature responses, the SE module emphasizes discriminative information while suppressing redundant features. Overall, the framework comprises two parallel branches: a convolutional branch for extracting two-dimensional spatial features and a temporal branch for modeling sequential dependencies. Feature integration and final classification are performed in the fusion layer. In particular, the architecture exploits the complementary strengths of CNN and LSTM: the CNN branch focuses on local spatial patterns under SE-enhanced channel dependencies, whereas the LSTM branch captures long-range temporal correlations. The overall network structure is shown in Figure 8, and each component is described in detail in the following sections.

#### 2.3.1 Squeeze-and-excitation block

**Figure 9:**
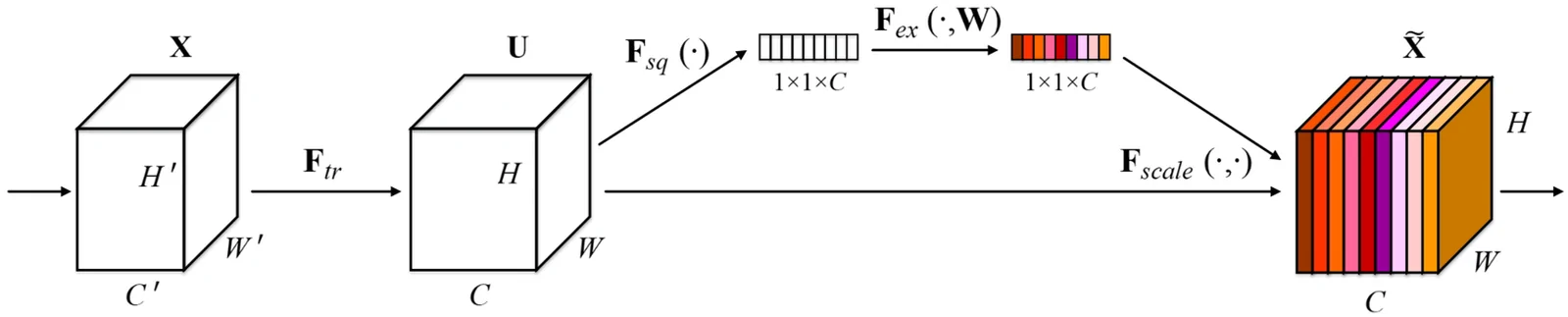
Squeeze and excitation block.

Channel attention aims to adaptively recalibrate the importance of each feature channel, automatically determining which information should be emphasized. In neural networks, different feature-map channels typically encode distinct types of information. Hu et al. (28) first introduced the concept of channel attention and proposed the squeeze-and-excitation (SE) module to implement it, as illustrated in Figure 9. The core idea of the SE module is to explicitly model inter-channel dependencies and adaptively reweight feature channels according to their relevance, thereby enhancing the network’s representational capacity. The module consists of two main operations: squeeze and excitation. The squeeze operation aggregates global spatial information through global average pooling to generate a compact channel descriptor. The excitation operation then employs fully connected layers with nonlinear activations to capture inter-channel dependencies and produce normalized attention weights. Finally, each channel of the input feature map is rescaled by its corresponding attention weight, achieving adaptive feature recalibration. Given an input feature tensor $X\in R^{C\times H\times W}$, the SE channel attention mechanism is mathematically expressed in Equation (10):

$$a=\sigma \left(\Theta_{2}\;\delta (\Theta_{1}\;\mathrm{GAP}(X))\right),\;Y=a⊙X$$ (10)

where GAP(⋅) denotes global average pooling, $a\in R^{C}$ is the channel attention vector, and Y is the recalibrated feature map. $\Theta_{1}\in R^{(C/r)\times C}$ and $\Theta_{2}\in R^{C\times (C/r)}$ are the weights of the two fully connected layers, r is the channel reduction ratio, δ(⋅) and σ(⋅) denote the ReLU and sigmoid activations, respectively, and ⊙ represents channel-wise multiplication.

In the CNN branch of this study, a squeeze-and-excitation (SE) module is inserted after each of the first two convolutional layers to adaptively reweight feature channels. This mechanism allows the network to amplify discriminative spectral–spatial information while suppressing redundant responses, thereby strengthening feature representation and improving both classification accuracy and generalization performance.

#### 2.3.2 CNN branch architecture

In recent years, CNN models have remained important architectures in deep learning and have demonstrated strong performance on data with large scale and high dimensionality. As a hierarchical feature extractor, a CNN learns discriminative patterns via local convolutions with shared weights, together with nonlinear activations for powerful feature mapping. A typical CNN comprises convolution, activation (such as ReLU), pooling, and fully connected layers, where pooling reduces feature resolution and improves invariance, and the final layers aggregate high-level representations for classification or regression.

**Figure 10:**
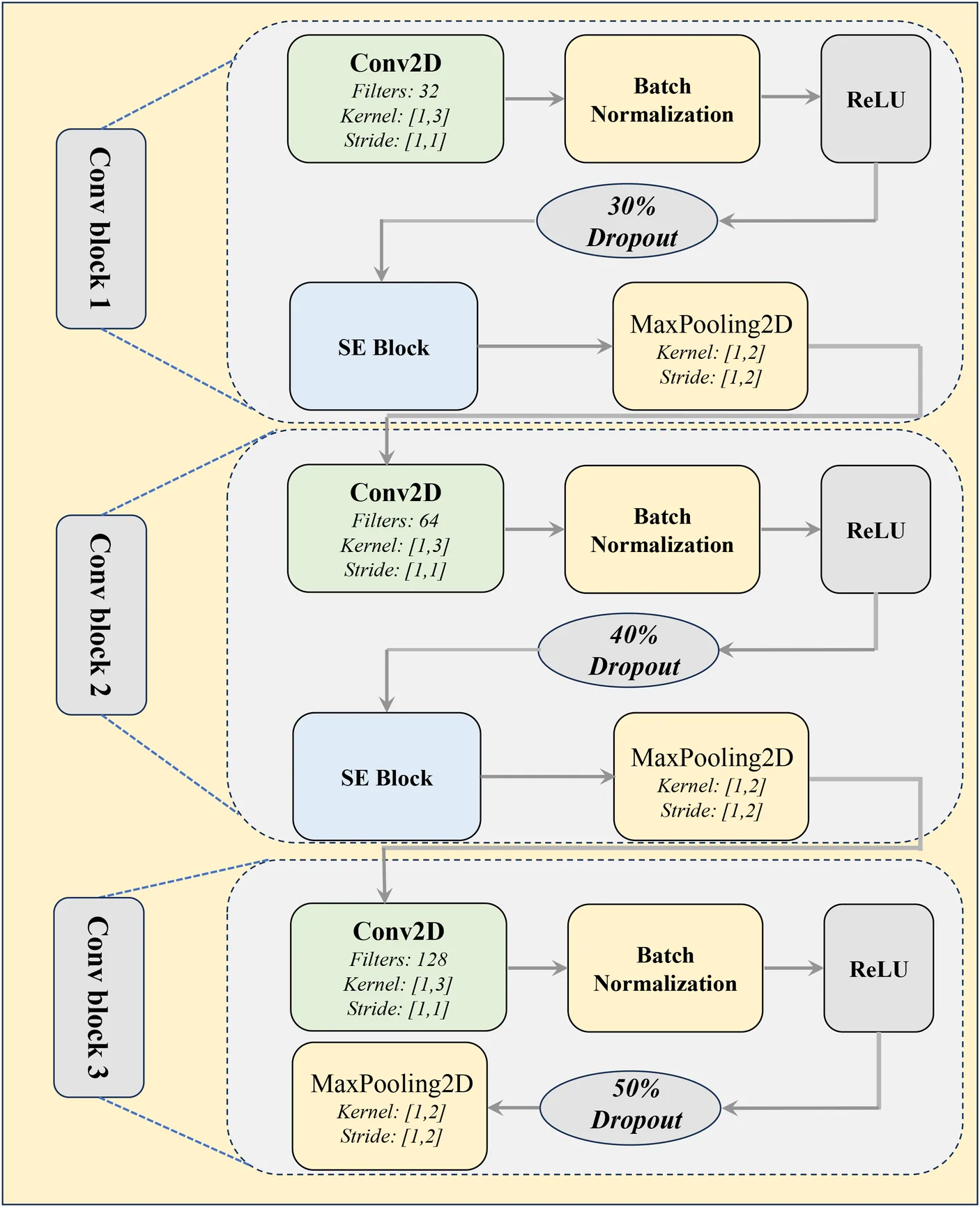
CNN branch architecture of HSD-Net.

The CNN branch of the proposed HSD-Net (Figure 10) comprises three convolutional layers, forming a hierarchical CNN branch that progressively extracts local features. The first convolutional layer uses 32 kernels within local receptive fields to capture basic representations of the input data. Its output is passed through batch normalization (BN) to stabilize training and accelerate convergence, followed by a ReLU activation function to introduce nonlinearity and enhance feature expressiveness. A squeeze-and-excitation (SE) module is then applied to adaptively recalibrate channel-wise weights, thereby highlighting informative feature channels. Finally, a max-pooling layer downsamples the feature maps, reducing dimensionality and improving translation invariance. The second convolutional block follows the same structure but increases the number of kernels to 64 to enable deeper feature extraction. After BN, ReLU, and SE, another max-pooling layer is applied for further feature compression. In the third convolutional block, the number of kernels is increased to 128 to capture higher-level abstract representations. Following BN and ReLU activation, a spatial dropout layer is introduced to mitigate overfitting by randomly dropping entire feature maps during training. A global average pooling layer is then used to aggregate the extracted features, yielding a compact representation for subsequent temporal feature fusion.

#### 2.3.3 BiLSTM branch architecture

RNN models are designed to process sequential data and have been widely used in speech recognition, image captioning, and language modeling. However, standard RNN models often suffer from vanishing and exploding gradients during training and have limited ability to capture long range dependencies; in long or noisy sequences, early informative cues may be gradually diluted by later inputs, degrading convergence stability and generalization. To mitigate these issues, Hochreiter and Schmidhuber (29) proposed long short-term memory (LSTM), which introduces memory cells and gating mechanisms to selectively retain, update, and output information, thereby stabilizing gradient flow and modeling both short- and long-term dependencies.

An LSTM consists of a forget gate $f_{t}$, an input gate $i_{t}$, and an output gate $o_{t}$, which regulate how much of the previous cell state $c_{t-1}$ is retained, what new information is written into the memory, and how much of the updated state is exposed as the hidden output $h_{t}$. These gates typically produce coefficients in [0,1] via the sigmoid function σ(⋅), enabling selective information flow and reducing the influence of irrelevant or noisy content. Some variants further incorporate peephole connections, allowing the gates to directly access the cell state $c_{t}$ for finer temporal control.

The primary output C at time step t is formulated in Equation (11):

$$C_{t}=f_{t}C_{t-1}+i_{t}c_{t}$$ (11)

Here, $f_{t}$ denotes the activation of the forget gate, $i_{t}$ represents the activation of the input gate, and $c_{t}$ denotes the cell state at time step t. The gate activations are obtained by applying the sigmoid function σ(⋅), whereas the hidden state is computed by passing the cell state through the hyperbolic tangent function tanh(⋅). The hidden state is updated according to Equation (12):

$$h_{t}=o_{t}⊙\tanh\;(c_{t}),$$ (12)

where $h_{t}$ is the hidden state at time step t, $o_{t}$ is the output gate activation, and ⊙ denotes element-wise multiplication.

The mathematical expressions for all gates are formulated in Equations (13)–(16):

$$i_{t}=\sigma \left(W_{xi}x_{t}+W_{hi}h_{t-1}+W_{ci}c_{t-1}+b_{i}\right)$$ (13)

$$f_{t}=\sigma \left(W_{xf}x_{t}+W_{hf}h_{t-1}+W_{cf}c_{t-1}+b_{\;f}\right)$$ (14)

$$o_{t}=\sigma \left(W_{xo}x_{t}+W_{ho}h_{t-1}+W_{co}c_{t}+b_{o}\right)$$ (15)

$$c_{t}=\tanh\;\left(W_{xc}x_{t}+W_{hc}h_{t-1}+b_{c}\right)$$ (16)

where $W_{x*}$ are the input-to-gate weights, $W_{h*}$ are the hidden-to-hidden weights, and $W_{c*}$ are the peephole weights.

To further exploit temporal dependencies, we adopt a Bidirectional Long Short-Term Memory (BiLSTM) network. A BiLSTM processes the input sequence in both forward and backward directions using two parallel LSTM layers, enabling the model to leverage both past and future contextual information for sequence representation. In this work, the outputs from the two directions are concatenated and fed into subsequent layers for downstream classification. This study adopts a two-layer sequential BiLSTM structure: the first layer contains 32 units per direction, totaling 64 units in both forward and backward directions, while the second layer includes 64 units per direction, totaling 128 units during training.

#### 2.3.4 Dual-branch fusion and output dimensionality

A dual-branch architecture with a CNN branch and a BiLSTM branch is adopted to capture localized spectrotemporal patterns and long-range temporal dependencies in heart sound features. The input is a frame-wise feature matrix of size T × 52, where T denotes the number of frames and 52 denotes the feature dimension per frame. For the CNN branch, the input is expanded by a singleton channel dimension to form T × 52 × 1 under the channels-last format, while the BiLSTM branch directly takes the sequence input of size T × 52. In the CNN branch, three convolutional blocks are used, each consisting of a 1 × 3 convolution with same padding followed by batch normalization and ReLU activation. The first block produces T × 52 × 32, followed by an SE module and 1 × 2 max pooling along the feature dimension to obtain T × 26 × 32. The second block produces T × 26 × 64, and the same SE and 1 × 2 max pooling yield T × 13 × 64. The third block produces T × 13 × 128, after which spatial dropout is applied. A global average pooling layer then aggregates the feature map into a 128-dimensional sample-level embedding. In the BiLSTM branch, two stacked bidirectional LSTM layers are employed for temporal modeling. The first layer uses 32 units per direction and outputs a sequence of size T × 64. The second layer uses 64 units per direction and outputs a sequence of size T × 128. Temporal global average pooling is finally applied to produce a 128-dimensional sample-level embedding.

For fusion and classification, the two 128-dimensional embeddings are first aligned by projecting each embedding to 32 dimensions using fully connected layers. The aligned vectors are concatenated along the feature dimension to form a 64-dimensional fused vector. This fused vector is further transformed by a fully connected fusion layer into a 64-dimensional representation and regularized by dropout. A fusion-level SE module is then applied to recalibrate the fused channels. Using a reduction ratio of 8, the module generates channel-wise weights and rescales the fused representation through element-wise multiplication, while preserving the 64-dimensional shape. Finally, the recalibrated vector is fed into the classification head, which maps it to 32 dimensions with dropout and produces the final two-class probability output via a two-unit fully connected layer with Softmax. For clarity and reproducibility, the layer-wise operations and corresponding output dimensionalities of both branches and the fusion head are summarized in Table 3.

**Table 3:** Layer-wise operations and corresponding output dimensionalities of the HSD-Net dual-branch CNN-BiLSTM architecture (T: number of frames).

Module	Operation	Output shape
Input	Feature sequence	T×52
CNN branch		
CNN-In	Reshape	T×52×1
Block 1	Conv(32) + SE + Pool	T×26×32
Block 2	Conv(64) + SE + Pool	T×13×64
Block 3	Conv(128) + Dropout	T×13×128
CNN-GAP	Global average pooling	128
BiLSTM branch		
LSTM-In	Sequence input	T×52
BiLSTM 1	32 units/direction	T×64
BiLSTM 2	64 units/direction	T×128
LSTM-GAP	Temporal average pooling	128
Fusion and classification		
Align	FC projection (128→32) ×2	32+32
Concat	Concatenation	64
Fusion	FC(64→64) + Dropout + SE	64
Head	FC(64→32) + Dropout	32
Output	FC(32→2) + Softmax	2

## 3 Experiments and results

Four groups of experiments are carried out to evaluate the classification performance of HSD-Net for cardiac disease screening. Specifically, the first group evaluates the impact of different acoustic feature configurations; the second assesses performance under alternative network connection strategies; the third applies the optimal feature representation and network configuration to an additional publicly available five-class heart sound dataset; and the fourth benchmarks the proposed HSD-Net against conventional classifiers and advanced methods previously reported in the literature.

### 3.1 Experiment setup

This experiment was conducted in the following software and hardware environment: the processor is an Intel® Core™ i5-14600KF (3.50 GHz), equipped with an NVIDIA RTX 4070 graphics card, and the operating system is Windows 11 Professional. All signal loading, preprocessing, and cepstral feature extraction (MFCC/IMFCC and their delta coefficients), as well as the conversion of the processed signals into model-ready input formats, were implemented in Python using Librosa (0.11.0) and SciPy (1.13.1). The deep learning framework used is TensorFlow 2.10.0 (CUDA 11.8), running in a Python 3.9.21 environment. The batch size for model training was set to 32, and the model was trained for a maximum of 100 epochs. The Adam optimizer was used during training, with an initial learning rate of 0.001. The Adam moment coefficients $\left(\beta_{1},\beta_{2}\right)$ were set to 0.9 and 0.999, respectively.

### 3.2 Training and evaluation metrics

The training process was based on a 10-fold cross-validation scheme and used the adaptive moment estimation (Adam) optimizer for parameter updates. The entire dataset was stratified by class and randomly partitioned into 10 mutually exclusive folds. In each iteration, 9 folds were used for training and the remaining fold was reserved for testing. This procedure was repeated until every fold had served exactly once as the test set, ensuring that all samples contributed to both training and evaluation. For each training split, 10% of the samples were further randomly selected from the training set to form a validation set, which was used to monitor convergence, trigger early stopping, and adaptively adjust the learning rate. Early stopping was performed by monitoring the validation loss, with the patience parameter set to 10 epochs. A ReduceLROnPlateau strategy was adopted for learning-rate adjustment. Specifically, the validation loss was monitored, and the learning rate was reduced by a factor of 0.5 when no improvement was observed for 10 consecutive epochs. To alleviate class imbalance during training, class weights were computed from the class distribution of the inner training subset after the validation split within each cross-validation iteration and incorporated into the loss function during model optimization. The validation subset and the held-out test fold were not used for class-weight estimation. The loss function was categorical cross entropy with label smoothing. Training hyperparameters included a batch size of 32, a maximum of 100 epochs, an L2 regularization coefficient of 0.0001, and an initial learning rate of 0.001.

For the evaluation of the proposed method, four metrics were used in each experiment: Accuracy, Recall, Precision, and F1-score. These metrics are defined in Equations (17)–(22):

$$\text{Accuracy}=\frac{\mathrm{TP}+\mathrm{TN}}{\mathrm{TP}+\mathrm{TN}+\mathrm{FP}+\mathrm{FN}}$$ (17)

$$\text{Sensitivity}=\frac{\mathrm{TP}}{\mathrm{TP}+\mathrm{FN}}$$ (18)

$$\text{Specificity}=\frac{\mathrm{TN}}{\mathrm{TN}+\mathrm{FP}}$$ (19)

$$\text{Precision}=\frac{\mathrm{TP}}{\mathrm{TP}+\mathrm{FP}}$$ (20)

$$\text{Recall}=\frac{\mathrm{TP}}{\mathrm{TP}+\mathrm{FN}}$$ (21)

$$F1=2\cdot \frac{\text{Precision}\times \text{Recall}}{\text{Precision}+\text{Recall}}$$ (22)

where TP denotes the number of true positive results, TN denotes the number of true negative results, FP denotes the number of false positive results, and FN denotes the number of false negative results.

### 3.3 Performance of hybrid cepstral fusion features

To verify the effectiveness of the proposed fusion features, four feature configurations were designed: (a) MFCC, (b) MFCC + ΔMFCC, (c) MFCC + IMFCC, and (d) the proposed hybrid cepstral fusion features (MFCC, IMFCC and their first-order delta coefficients). A control condition using IMFCC alone was not included. IMFCC is highly effective at capturing high-frequency pathological details in heart sounds but is less suitable for representing low-frequency fundamental components. These low-frequency components, which encode the normal cardiac cycle and key valvular acoustic characteristics, are essential for a complete description of the signal. Each feature configuration was trained using the same neural network architecture and hyperparameters, and the final performance was reported as the average of the evaluation metrics across the 10 cross-validation folds. Considering that the energy of some pathological murmurs may extend to higher frequency bands up to 600–800 Hz, we investigated the influence of filter bandwidth on feature preservation and classification performance. Specifically, while keeping all other settings unchanged, we repeated the experiments by widening the Butterworth bandpass range from 20–200 Hz to 20–400 Hz, 20–600 Hz, 20–700 Hz, and 20–800 Hz. Table 4 summarizes the results of the proposed hybrid cepstral fusion features under different passbands. Under identical settings, the proposed fusion features achieve better overall classification performance than the single or partially fused feature configurations. Moreover, expanding the passband to retain more high-frequency components improves performance up to the 20–600 Hz setting, which achieves the highest sensitivity and accuracy. Although the 20–700 Hz setting yields slightly higher specificity and precision, further increasing the upper cutoff beyond 600 Hz does not lead to additional accuracy gains. Therefore, considering the overall balance among the evaluation metrics and the screening-oriented objective of this study, the 20–600 Hz setting was selected as the final configuration. This finding suggests that preserving frequency components up to 600 Hz is sufficient to provide the high-frequency cues required for effective IMFCC analysis, whereas further increasing the upper cutoff does not lead to additional performance gains.

**Table 4:** Classification performance of different feature configurations and hybrid cepstral fusion under varied bandpass settings.

Feature type	Accuracy	Sensitivity	Specificity	Precision
MFCC	93.95% ± 0.49%	95.23% ± 0.76%	93.17% ± 0.58%	89.46% ± 0.92%
MFCC+ΔMFCC	94.67% ± 0.45%	94.13% ± 0.82%	95.00% ± 0.52%	91.98% ± 0.86%
MFCC+IMFCC	95.09% ± 0.40%	93.77% ± 0.74%	95.89% ± 0.47%	93.29% ± 0.76%
Hybrid cepstral features				
(20–200 Hz)	95.65% ± 0.41%	94.80% ± 0.71%	96.35% ± 0.46%	94.25% ± 0.76%
(20–400 Hz)	95.92% ± 0.38%	94.95% ± 0.69%	96.60% ± 0.44%	94.70% ± 0.73%
**(20–600 Hz)**	**96.25% ± 0.32%**	**95.85% ± 0.58%**	**96.50% ± 0.36%**	**94.41% ± 0.62%**
(20–700 Hz)	96.15% ± 0.35%	95.20% ± 0.63%	96.80% ± 0.43%	94.75% ± 0.69%
(20–800 Hz)	96.13% ± 0.35%	95.10% ± 0.64%	96.78% ± 0.44%	94.70% ± 0.70%

Bold values indicate the best performance.

**Figure 11:**
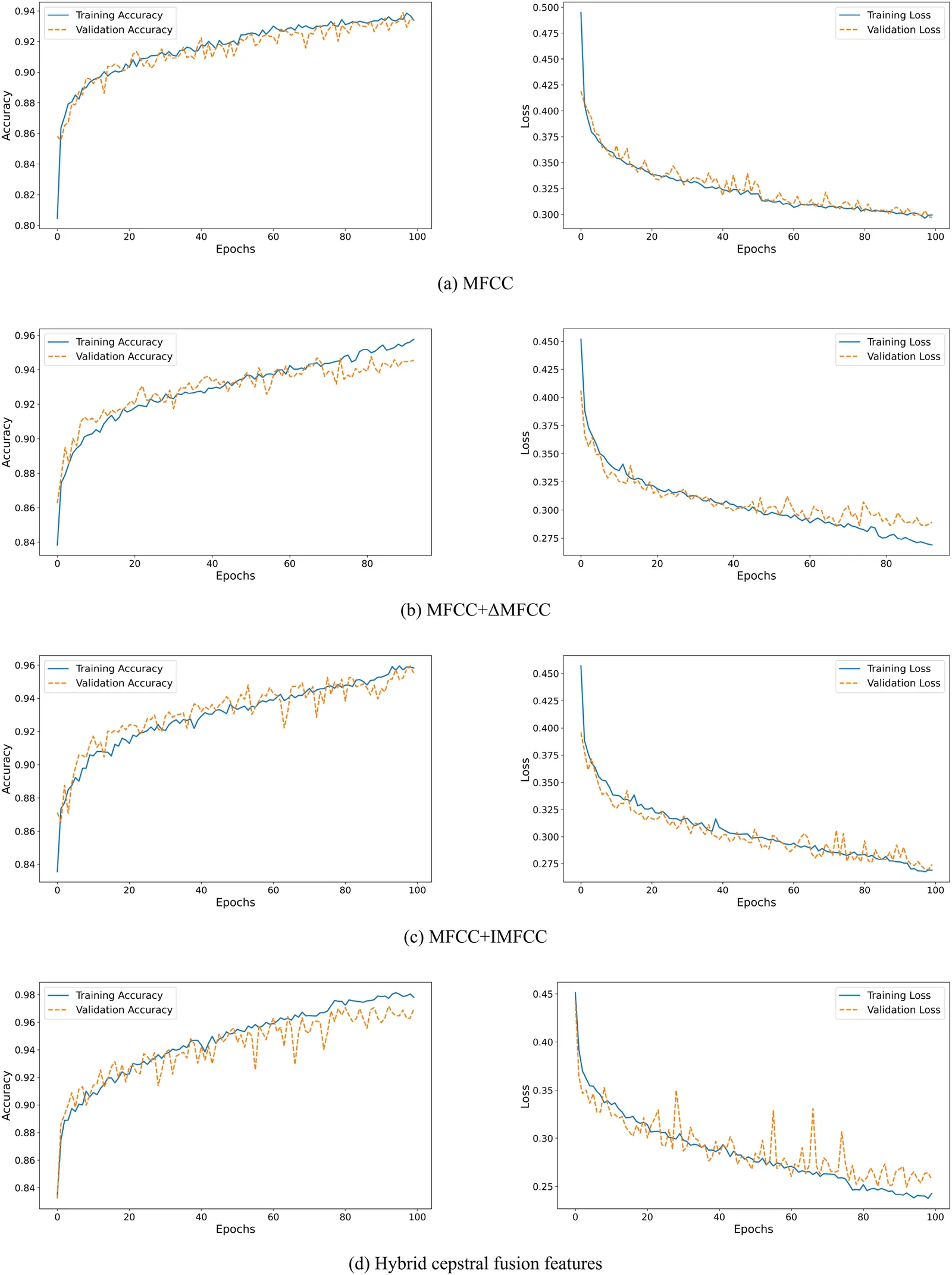
Comparison of training and validation accuracy and loss across different feature types on the PhysioNet/CinC Challenge 2016 dataset: (**a**) MFCC; (**b**) MFCC + ΔMFCC; (**c**) MFCC + IMFCC; (**d**) hybrid cepstral fusion features.

The corresponding training and validation accuracy and loss curves are shown in Figure 11. Compared with MFCC alone, adding ΔMFCC leads to slightly faster convergence and a modest improvement in validation accuracy, indicating that temporal derivatives provide useful dynamic information beyond static spectral envelopes. When IMFCC is introduced together with MFCC, the curves further improve and converge to a higher accuracy level, suggesting that the additional high-frequency emphasis helps capture pathological acoustic details that are less effectively represented by MFCC alone. The proposed hybrid cepstral fusion features achieve the best final training performance and the highest overall validation accuracy, although the validation curve exhibits somewhat larger fluctuations during training. We attribute this behavior to the richer and more sensitive joint representation formed by combining low-frequency MFCC information, high-frequency IMFCC information, and their temporal dynamics, which enhances discriminative power but also makes the validation process more responsive to sample variation. Overall, these results indicate that progressively incorporating dynamic information and frequency band complementarity improves feature quality and ultimately leads to better classification performance.

### 3.4 Performance of the HSD-Net dual-branch architecture

To evaluate the classification performance of the model, we applied the proposed algorithm to multiple deep learning architectures. The baseline models included (a) CNN and (b) BiLSTM. To verify the effectiveness of the dual-branch design, we further implemented (c) a CNN–BiLSTM single-branch sequential fusion model and compared it with (d) the CNN–BiLSTM dual-branch model. The experimental results are summarized in Table 5, while Table 6 reports the architectural details, parameter counts, and mean inference latency of each evaluated model, with the latency averaged over 10 repeated inference runs. As shown in Table 6, the proposed HSD-Net contains 136,790 trainable parameters and achieves a mean inference latency of approximately 43 ms per input segment under the experimental hardware environment.

**Table 5:** Classification performance of different network architectures.

Model	Accuracy	Sensitivity	Specificity	Precision
CNN	93.89% ± 0.51%	93.16% ± 0.92%	94.34% ± 0.50%	90.89% ± 0.95%
BiLSTM	93.95% ± 0.49%	93.53% ± 0.93%	94.20% ± 0.51%	90.73% ± 0.92%
HSD-Net (single branch)	94.67% ± 0.37%	92.21% ± 0.65%	96.17% ± 0.42%	93.58% ± 0.70%
**HSD-Net (dual branch)**	**96.25% ± 0.32%**	**95.85% ± 0.58%**	**96.50% ± 0.36%**	**94.41% ± 0.62%**

Bold values indicate the best performance.

**Table 6:** Architectural parameters and mean inference latency of evaluated models.

Model	Structure details	Params	Inference time
(a) CNN	Conv[32,(1,3)]-BN-ReLU-SE-MaxPooling(1,2)-Conv[64,(1,3)]-BN-ReLU-SE-MaxPooling(1,2)-Conv[128,(1,3)]-BN-ReLU-Dropout(0.3)-GlobalAveragePooling()-Dense(32)-Drop(0.4)-SE-Dense(32)-Drop(0.3)-Dense(2)	38,866	31 (ms)
(b) BiLSTM	BiLSTM(32)-Drop(0.3)-BiLSTM(64)-Drop(0.3)-GlobalAverage Pooling1D()-Dense(32)-Drop(0.3)-Dense(2)	92,002	35 (ms)
(c) CNN–BiLSTM	Conv[32,(1,3)]-BN-ReLU-SE-MaxPooling(1,2)-Conv[64,(1,3)]-BN-ReLU-SE-MaxPooling(1,2)-Conv[128,(1,3)]-BN-ReLU-Dropout(0.3)-Flatten-BiLSTM(32)-Drop(0.3)-BiLSTM(64)-Drop(0.3)-GlobalAveragePooling1D()-Dense(32)-Drop(0.3)-Dense(2)	537,998	26 (ms)
(d) HSD-Net	Conv[32,(1,3)]-BN-ReLU-SE-MaxPooling(1,2)-Conv[64,(1,3)]-BN-ReLU-SE-MaxPooling(1,2)-Conv[128,(1,3)]-BN-ReLU-Dropout(0.3)-GlobalAveragePooling();BiLSTM(32)-Drop(0.3)- BiLSTM(64)-Drop(0.3)-GlobalAveragePooling1D();Dense(32)-Dense(32)-Concat-Dense(64)-Drop(0.4)-SE-Dense(32)-Drop(0.3)-Dense(2)	136,790	43 (ms)

**Figure 12:**
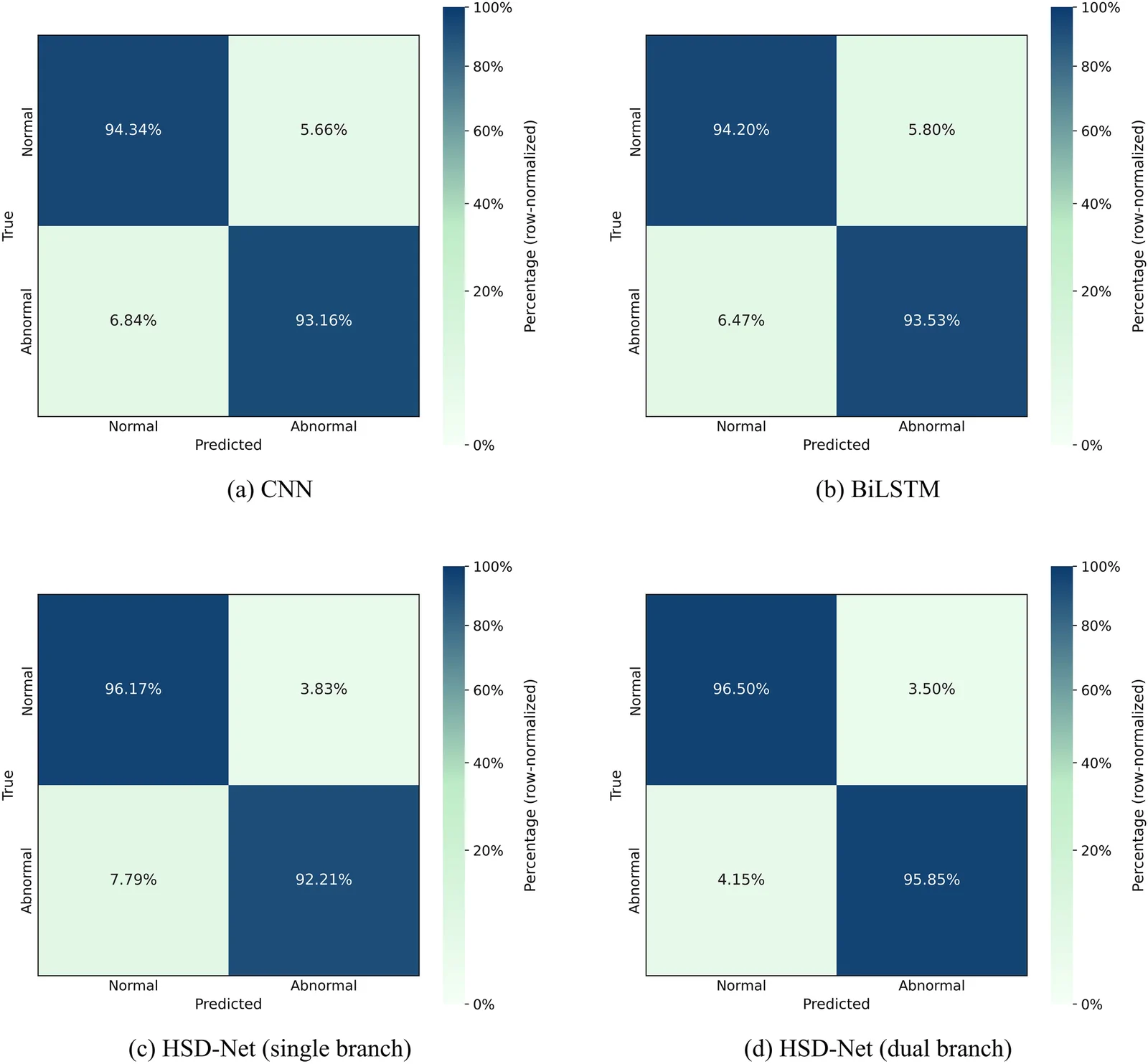
Row-normalized confusion matrices (%) of different models on the PhysioNet/CinC Challenge 2016 dataset: **(a)** CNN, **(b)** BiLSTM, **(c)** HSD-Net (single branch), and **(d)** HSD-Net (dual branch).

**Figure 13:**
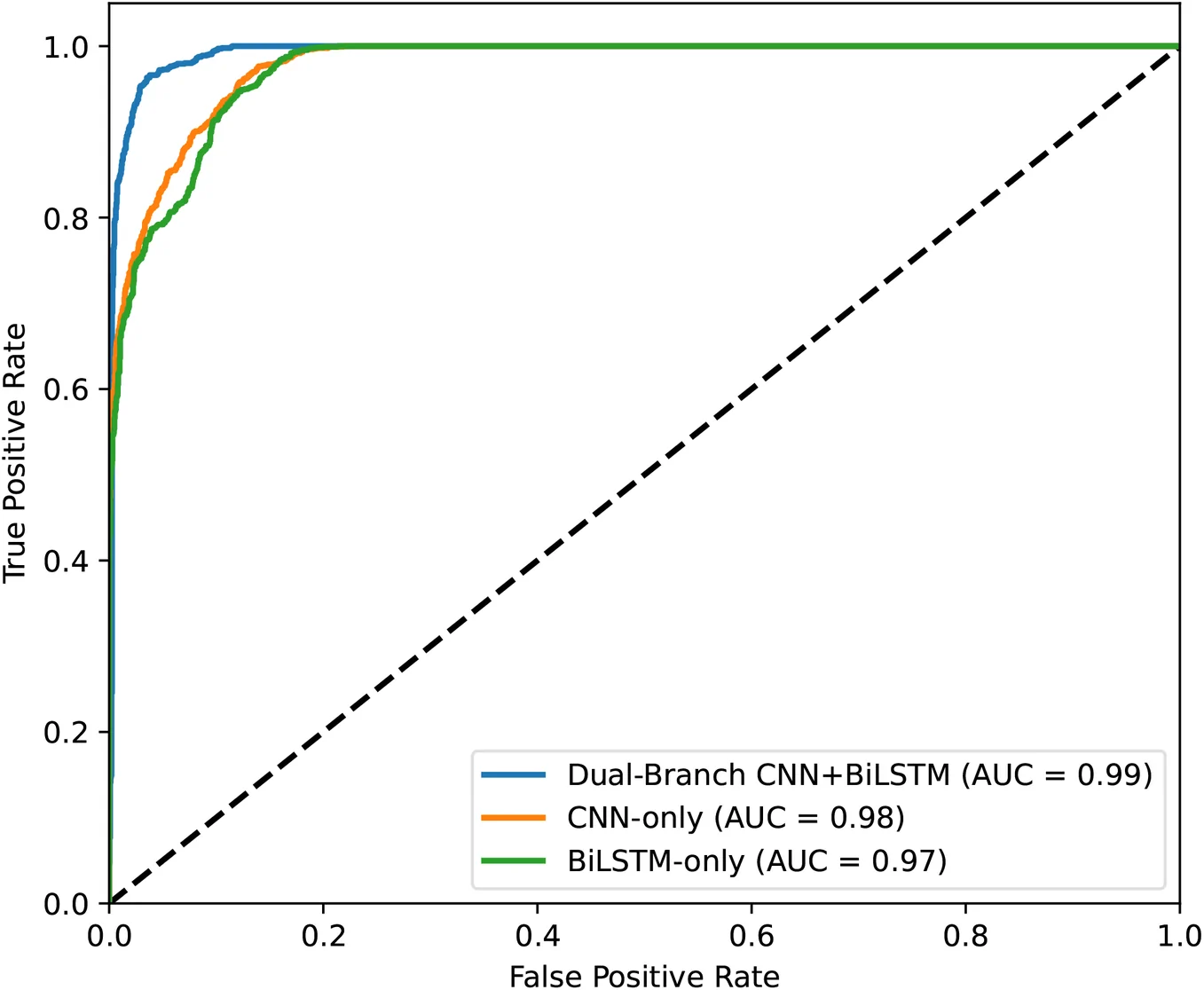
ROC curves and AUC scores of different models on the PhysioNet/CinC challenge 2016 dataset.

In this study, the PhysioNet/CinC Challenge 2016 recordings were analyzed using 4 s fixed-length windows. The inference latency is substantially shorter than the duration of the analysis window. This result indicates that the proposed model has sufficient computational efficiency for heart sound screening in real time or near real time during model inference. The two baseline models achieve comparable performance, with CNN achieving an accuracy of 93.89% and BiLSTM achieving an accuracy of 93.95%. Building on this, the single-branch fusion model further improves the performance, reaching an accuracy of 94.67%. The dual-branch HSD-Net achieves the best overall results across all metrics, with sensitivity 95.85%, specificity 96.50%, precision 94.41%, and accuracy 96.25%, indicating that the dual-branch architecture more effectively integrates complementary representations and thus enhances classification performance. To provide a more intuitive view of the classification outcomes and detailed accuracy, we report the confusion matrices of the classification results for each model in Figure 12. In addition, to assess the overall discrimination ability under different decision thresholds, we further plot the receiver operating characteristic (ROC) curves. As shown in Figure 13, the dual-branch CNN-BiLSTM achieves an AUC of 0.99, which is higher than those of the single CNN and single BiLSTM models. Moreover, its ROC curve remains consistently closer to the upper-left corner, indicating a more favorable trade-off between true positive rate and false positive rate across a wide range of thresholds. Although the single-branch models also exhibit strong discrimination ability, their curves are slightly farther from the ideal boundary. These results demonstrate that the proposed dual-branch architecture can better exploit the complementary spatial and temporal information in heart sound signals, thereby yielding stronger overall discrimination performance on the PhysioNet/CinC Challenge 2016 dataset.

Building on this, to verify the advantage of BiLSTM over unidirectional LSTM and gated recurrent unit (GRU) within the fusion architecture, we replaced the BiLSTM branch in the original fusion model with a unidirectional LSTM and a GRU, respectively. To ensure a fair comparison, all other network components and training settings were kept unchanged. The experimental results reported in Table 7 show that the BiLSTM-based configuration consistently achieves the best performance, confirming its superior ability to model bidirectional cardiac-cycle-level temporal dependencies. This result is as expected, since BiLSTM can model bidirectional temporal information, whereas unidirectional LSTM and GRU have more limited temporal modeling capability. This indicates that bidirectional temporal encoding is more effective for capturing subtle variations in heart sound signals.

**Table 7:** Classification performance of HSD-Net with different recurrent units.

Model (dual branch)	Accuracy	Sensitivity	Specificity	Precision
CNN–LSTM	94.22% ± 0.40%	93.20% ± 0.69%	94.85% ± 0.44%	91.73% ± 0.79%
CNN–GRU	94.67% ± 0.39%	93.53% ± 0.72%	95.80% ± 0.45%	93.58% ± 0.78%
**HSD-Net (dual branch)**	**96.25% ± 0.32%**	**95.85% ± 0.58%**	**96.50% ± 0.36%**	**94.41% ± 0.62%**

Bold values indicate the best performance.

Finally, to assess the role of the squeeze-and-excitation (SE) module in the proposed fusion architecture, we designed four ablation configurations under identical data partitioning, feature extraction, and training procedures: (a) no SE module (baseline); (b) SE applied only to the CNN branch; (c) SE applied only to the fusion layer; and (d) SE applied simultaneously to both the CNN branch and the fusion layer. Apart from the presence or absence of the SE module, the network depth, number of channels, and all hyperparameters were kept unchanged. The experimental results shown in Table 8 indicate that SE-based configurations generally improve classification performance compared with the baseline model. Applying SE to either the CNN branch or the fusion layer alone improves the overall accuracy, while the configuration (d) integrating SE modules in both components achieves the optimal overall performance. This finding suggests that SE modules enhance channel-wise feature recalibration, and their synergistic application in both branch-level extraction and fusion-level integration further improves the model’s classification performance.

**Table 8:** Performance of HSD-Net with different SE module configurations.

Configuration	Accuracy	Sensitivity	Specificity	Precision
No SE (baseline)	94.28% ± 0.43%	92.90% ± 0.74%	95.20% ± 0.48%	92.80% ± 0.83%
SE on CNN branch only	94.75% ± 0.41%	93.88% ± 0.69%	95.35% ± 0.47%	93.08% ± 0.75%
SE at fusion layer only	95.85% ± 0.36%	95.20% ± 0.70%	96.10% ± 0.42%	94.75% ± 0.69%
**SE on CNN branch + fusion layer**	**96.25% ± 0.32%**	**95.85% ± 0.58%**	**96.50% ± 0.36%**	**94.41% ± 0.62%**

Bold values indicate the best performance.

After determining the final HSD-Net configuration, we conducted a noise-injection experiment on the PhysioNet/CinC Challenge 2016 dataset to examine how the classification performance changed under additional acoustic perturbations. Specifically, noise samples from the MUSAN database (30) were added only to the held-out test folds at signal-to-noise ratio (SNR) levels of +20 dB, +10 dB, and +5 dB, while the training data remained unchanged. This experiment was not intended to evaluate an individual model component, but to provide an additional assessment of model performance under externally introduced noise. As shown in Table 9, the classification performance gradually decreased as the SNR level decreased, which is consistent with the increased difficulty of heart sound classification under noisier acoustic conditions. Nevertheless, HSD-Net still retained reasonable classification performance under the tested noise conditions, suggesting that the proposed model has a certain degree of resistance to acoustic noise.

**Table 9:** Performance comparison of HSD-Net on the PhysioNet/CinC Challenge 2016 dataset under clean and MUSAN-noise conditions at SNR levels of +20 dB, +10 dB, and +5 dB using 10-fold cross-validation.

SNR condition	Accuracy	Sensitivity	Specificity	Precision	F1-score
**Clean**	**96.25%±0.32%**	**95.85%±0.58%**	**96.50%±0.36%**	**94.41%±0.62%**	**95.12%±0.60%**
+20 dB	94.86%±0.58%	94.35%±0.72%	95.18%±0.55%	92.44%±0.80%	93.39%±0.76%
+10 dB	92.82%±0.84%	91.85%±0.95%	93.42%±0.78%	89.72%±1.02%	90.77%±0.98%
+5 dB	90.43%±1.12%	88.96%±1.20%	91.35%±1.05%	86.54%±1.28%	87.73%±1.23%

Bold values indicate the best performance.

### 3.5 Comparison with standard deep learning baselines using MFCC features

Although the preceding ablation experiments demonstrated the contributions of the hybrid cepstral features, the dual branch architecture, and the SE attention configuration within the proposed framework, they mainly focused on component level validation. Therefore, an additional comparison was conducted to further distinguish HSD-Net from standard deep learning pipelines using MFCC features, which have been widely adopted in previous heart sound classification studies. Conventional configurations, such as MFCC with CNN, MFCC with BiLSTM, and MFCC with sequential CNN and BiLSTM, represent typical feature and classifier pipelines for PCG classification. To examine whether the performance of HSD-Net can be explained merely by the use of MFCC features and conventional CNN or recurrent modules, several standard baselines using MFCC features were constructed and evaluated under the same preprocessing procedure, training strategy, and 10 fold cross validation protocol as the proposed model.

The baseline models included CNN, BiLSTM, and sequential CNN–BiLSTM models using MFCC features as input, which represent local feature learning, temporal dependency modeling, and sequential spatial temporal modeling, respectively. In addition, two dynamic MFCC based sequential CNN–BiLSTM baselines were included by adding first order delta coefficients and further adding second order delta coefficients to the MFCC input. These settings were used to examine whether conventional dynamic MFCC extensions were sufficient to approach the performance of HSD-Net. In contrast to these baselines, HSD-Net uses a hybrid cepstral representation that integrates MFCC, IMFCC, and their first order delta coefficients, and further processes this representation through a parallel CNN–BiLSTM architecture with SE based feature recalibration. The comparative results are summarized in Table 10.

**Table 10:** Comparison of HSD-Net with standard deep learning baselines using MFCC-based feature inputs on the PhysioNet/CinC Challenge 2016 dataset.

Feature input	Model	Accuracy	Sensitivity	Specificity
MFCC	CNN	92.37% ± 0.60%	89.60% ± 0.88%	94.10% ± 0.62%
MFCC	BiLSTM	92.97% ± 0.56%	90.45% ± 0.84%	94.55% ± 0.58%
MFCC	CNN–BiLSTM	93.47% ± 0.52%	91.25% ± 0.80%	94.85% ± 0.55%
MFCC + ΔMFCC	CNN–BiLSTM	93.82% ± 0.49%	91.85% ± 0.76%	95.05% ± 0.52%
MFCC + ΔMFCC + $\Delta^{2}$MFCC	CNN–BiLSTM	94.01% ± 0.47%	92.15% ± 0.74%	95.18% ± 0.50%
**Hybrid cepstral features**	**HSD-Net**	**96.25% ± 0.32%**	**95.85% ± 0.58%**	**96.50% ± 0.36%**

Bold values indicate the best performance.

As shown in Table 10, the standard baselines using MFCC features achieved competitive results, but their overall performance remained lower than that of the complete HSD-Net. The CNN and BiLSTM models using MFCC features provided separate references for convolutional local pattern extraction and recurrent temporal modeling, while the sequential CNN–BiLSTM model further combined these two modeling strategies. Although this sequential structure improved performance compared with the single model baselines, it still did not reach the performance of HSD-Net. Similarly, introducing first order and second order dynamic MFCC coefficients provided additional temporal variation information and further improved the sequential baseline, but the gap to the proposed method remained. These results suggest that the improvement of HSD-Net is not solely attributable to the reuse of MFCC features or conventional CNN and recurrent modules. Instead, it is more closely associated with the combined design of hybrid cepstral fusion and parallel feature integration, which jointly exploits low frequency cepstral information, high frequency complementary cues, and short term dynamic acoustic variations for heart sound classification.

### 3.6 Interpretability analysis with representative cases

**Figure 14:**
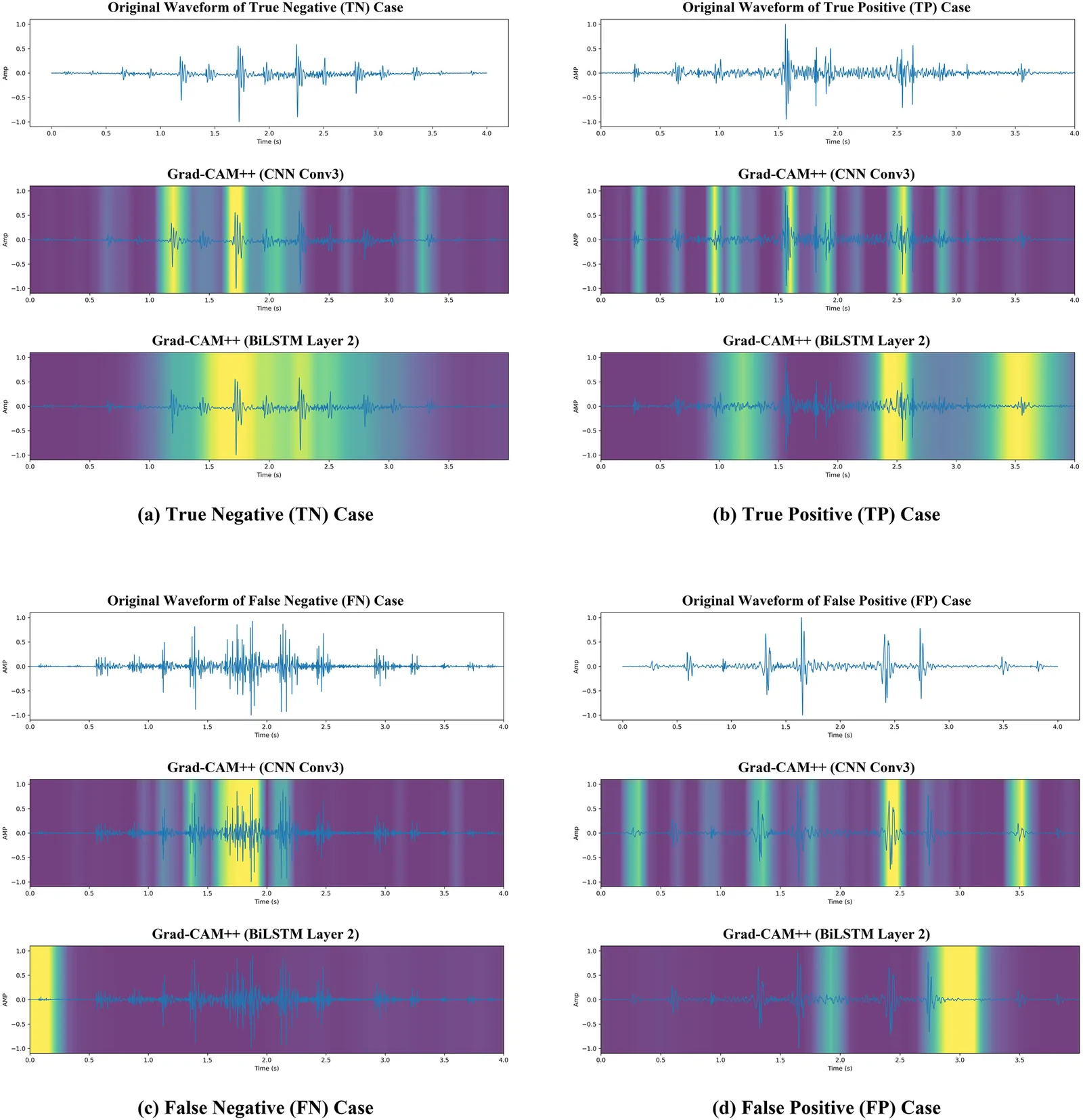
Original waveforms and 1D Grad-CAM++ saliency maps of HSD-Net's Conv3 and BiLSTM layer 2 for representative samples from the PhysioNet/CinC Challenge 2016 dataset: (**a**) true negative (TN); (**b**) true positive (TP); (**c**) false negative (FN); (**d**) false positive (FP).

To further elucidate the decision basis of the proposed model, we selected four representative samples for interpretability analysis, including a true negative (TN), a true positive (TP), a false negative (FN), and a false positive (FP). As shown in Figure 14, the original waveform and the Grad-CAM++ responses from the CNN branch (Conv3) and the BiLSTM branch (BiLSTM layer 2) were jointly examined for each case.

In the TN example, the high-response regions are mainly concentrated within several dominant heart sound activity windows, rather than being persistently activated throughout the entire signal. Specifically, the CNN branch shows localized strong responses around 1.15–1.28 s and 1.65–1.80 s, which largely coincide with the prominent high-amplitude heart sound events in the original waveform. Moderate responses are also observed around 1.95–2.05 s and 3.25–3.35 s, suggesting that the CNN branch primarily focuses on discrete acoustic events with clear waveform structures. Meanwhile, the BiLSTM branch forms a broader high-response region over approximately 1.45–2.35 s, with stronger emphasis around 1.55–1.85 s and 2.05–2.30 s. This indicates that the model not only attends to the dominant heart sound events themselves, but also incorporates their surrounding temporal context. Because the saliency remains mainly confined to the principal heart sound complexes and does not persist over the weak fluctuations before about 1.0 s or after about 2.8 s, the model does not appear to treat these nonspecific perturbations as abnormal evidence. This pattern supports the correct normal prediction with high confidence and suggests that the normal decision is primarily grounded in organized heart sound activity rather than scattered background fluctuations.

Compared with the TN example, the TP case exhibits a broader and more persistent saliency distribution, indicating that the model relies on abnormality-related evidence spanning multiple temporal intervals. Specifically, the CNN branch presents localized strong responses around 0.95–1.05 s, 1.55–1.65 s, and 2.50–2.60 s, which largely correspond to several prominent high-amplitude abnormal waveform events in the original signal. Meanwhile, the BiLSTM branch shows elevated responses over approximately 0.95–1.35 s, 2.45–2.65 s, and 3.35–3.70 s, with stronger emphasis around 2.50–2.60 s and 3.45–3.60 s. Unlike the TN pattern, which is mainly confined to principal normal heart sound events, the TP case shows a combined pattern of focal abnormal localization and sustained abnormal-context integration across the two branches, thereby providing more complete evidence for the correct abnormal classification. These highlighted intervals offer temporally traceable evidence for the abnormal decision and may also serve as useful references for targeted follow-up review.

In the FN example, the original waveform contains several prominent abnormal fluctuations, with the most intense activity concentrated approximately between 1.40–2.30 s and additional weaker abnormal events appearing later in the signal. The CNN branch shows a relatively strong and continuous high-response region over about 1.35–2.20 s, with the strongest emphasis around 1.55–1.95 s and secondary responses around 2.00–2.20 s. This indicates that the CNN branch is able to capture the main abnormal acoustic activity in the middle portion of the waveform. However, the BiLSTM branch does not provide corresponding temporal support for these abnormal intervals. Instead, its strongest response is concentrated mainly at the very beginning of the signal, approximately 0.00–0.20 s, while the subsequent abnormal regions receive only weak and diffuse attention. As a result, the abnormal evidence extracted by the CNN branch is not reinforced by the temporal branch, and the model fails to form sufficiently consistent cross-branch evidence for an abnormal decision, ultimately leading to a false negative prediction. This case suggests that when the temporal branch focuses on uninformative or weakly relevant segments rather than the principal abnormal activity, the overall model may underestimate the pathological significance of the sample.

In the FP example, the waveform is labeled as normal, yet the model incorrectly interprets several late-middle fluctuations as abnormal evidence. Specifically, the CNN branch shows strong localized responses primarily around 2.40–2.55 s and 3.45–3.55 s, with the most prominent activation occurring near 2.45–2.50 s, where the original waveform contains a sharp high-amplitude heart sound event. Meanwhile, the BiLSTM branch reaches its strongest response over approximately 2.75–3.25 s, suggesting that the temporal branch further regards the surrounding late-middle context as suspicious. When considered together with the original waveform, these highlighted regions mainly correspond to transient amplitude variations within an overall normal sample, rather than a persistent abnormal pattern. Thus, the misclassification in this case is likely caused by overinterpretation of several normal but salient waveform fluctuations as abnormal evidence. This observation indicates that highly activated local or temporal regions should still be interpreted in the context of the overall waveform pattern, rather than being regarded alone as sufficient evidence of abnormality.

From these representative cases, correct predictions generally coincide with coherent cross-branch evidence, whereas misclassifications often occur when abnormal cues are not sufficiently reinforced across branches or when salient but normal waveform variations are overemphasized. This suggests that the proposed model provides more than a normal/abnormal label. During prediction, saliency maps highlight the heart sound segments that contribute most strongly to the decision, helping clinicians prioritize intervals for re-auscultation and review. By comparing these high-response regions with the original waveform, clinicians can assess whether the model output is driven by clinically meaningful events or nonspecific fluctuations. Coherent and well-aligned branch responses may support targeted interpretation, whereas inconsistent, fragmented, or overly localized responses may indicate uncertainty and the need for further review. Overall, the proposed model can serve as an assisted tool for heart sound screening and clinical interpretation by localizing suspicious segments and providing reference information for follow-up assessment.

### 3.7 Ablation experiments on the Yaseen dataset

**Figure 15:**
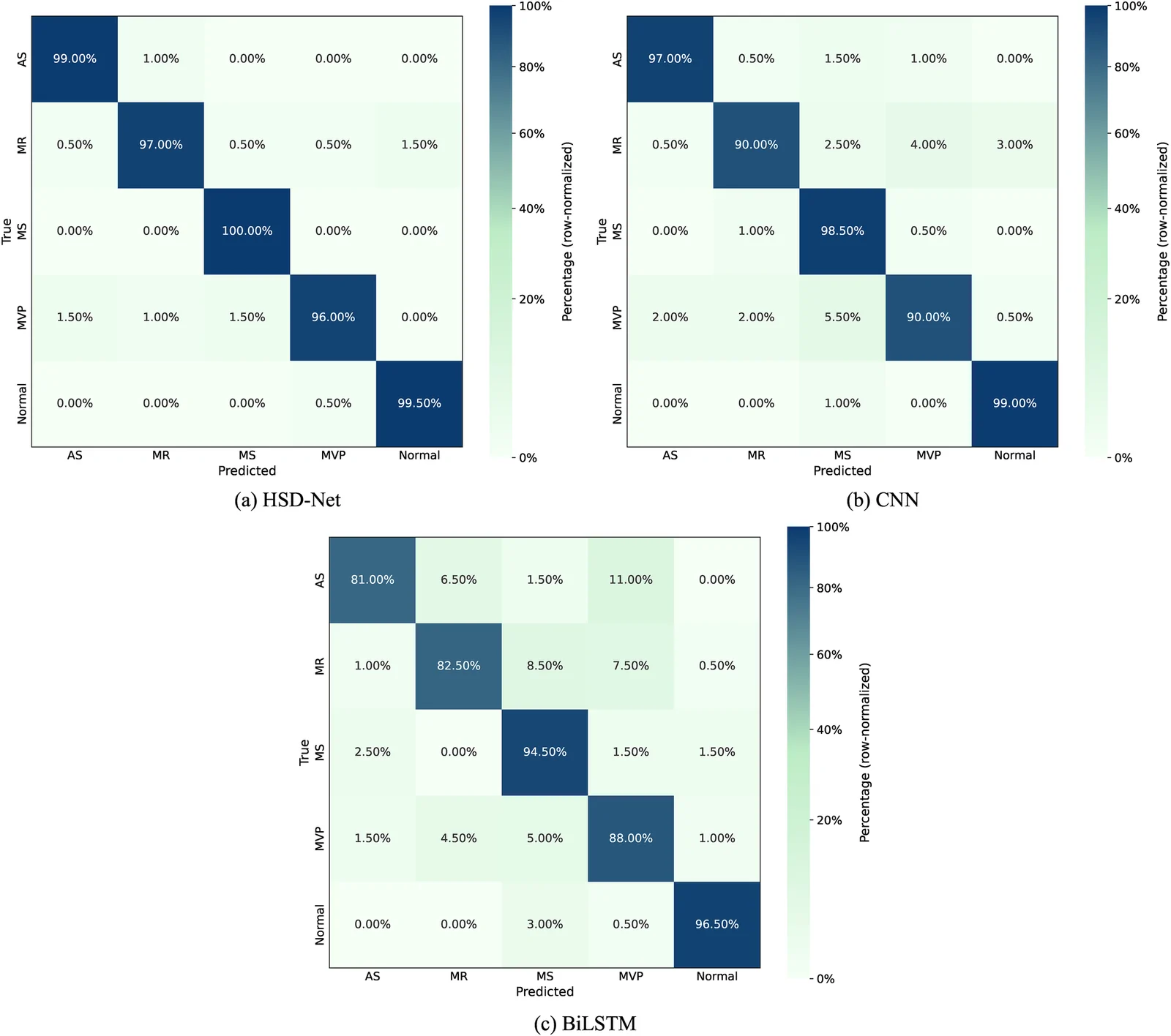
Row-normalized confusion matrices (%) for five-class valvular heart disease classification on the Yaseen dataset: **(a)** HSD-Net, **(b)** CNN, and **(c)** BiLSTM.

To validate the generalization capability of HSD-Net on an extra public dataset, we carry out ablation experiments based on the Yaseen benchmark. The experimental results are summarized in Tables 11–13, where Table 11 reports the performance of HSD-Net and Tables 12, 13 present the corresponding results of using the CNN and BiLSTM branches as standalone classifiers. As can be observed, HSD-Net consistently achieves higher overall accuracy and F1-scores than the single-branch models, confirming the advantage of the hybrid cepstral fusion and dual-branch design. The detailed classification outcomes are illustrated in Figure 15. As shown in the confusion matrices, HSD-Net exhibits a more concentrated diagonal distribution and higher per-class true positive percentages than the CNN-only and BiLSTM-only models, indicating fewer inter-class confusions among the five valvular heart sound categories.

**Table 11:** Comparison of per-class and macro-average performance of HSD-Net on the Yaseen dataset using 10-fold cross-validation.

Classes		Performance metrics				
		Accuracy	Sensitivity	Specificity	Precision	F1-score
	AS	99.40%±0.18%	99.00%±0.50%	99.50%±0.15%	98.05%±0.30%	98.51%±0.28%
	MR	99.00%±0.22%	97.00%±0.47%	99.50%±0.17%	98.06%±0.35%	97.50%±0.33%
	MS	99.60%±0.19%	100.00%±0.41%	99.50%±0.14%	98.12%±0.31%	99.03%±0.29%
	MVP	99.00%±0.21%	96.00%±0.46%	99.75%±0.18%	98.97%±0.33%	97.46%±0.31%
	Normal	99.60%±0.20%	99.50%±0.41%	99.62%±0.16%	98.52%±0.31%	99.01%±0.29%
**Average**		**99.32%±0.20%**	**98.30%±0.45%**	**99.57%±0.16%**	**98.34%±0.32%**	**98.30%±0.30%**

Bold values indicate the macro-average performance across all classes.

**Table 12:** Comparison of per-class and macro-average performance of the CNN branch classifier on the Yaseen dataset using 10-fold cross-validation.

Classes		Performance metrics				
		Accuracy	Sensitivity	Specificity	Precision	F1-score
	AS	98.90%±0.34%	97.00%±0.90%	99.37%±0.20%	97.50%±0.62%	97.21%±0.60%
	MR	97.30%±0.35%	90.00%±0.74%	99.12%±0.24%	96.30%±0.72%	93.03%±0.66%
	MS	97.60%±0.31%	98.50%±0.80%	97.37%±0.23%	90.44%±0.66%	94.27%±0.57%
	MVP	96.90%±0.33%	90.00%±0.86%	98.62%±0.19%	94.25%±0.70%	92.05%±0.61%
	Normal	99.10%±0.27%	99.00%±0.60%	99.12%±0.24%	96.61%±0.50%	97.78%±0.46%
**Average**		**97.96%±0.32%**	**94.90%±0.78%**	**98.72%±0.22%**	**95.02%±0.64%**	**94.87%±0.58%**

Bold values indicate the macro-average performance across all classes.

**Table 13:** Comparison of per-class and macro-average performance of the BiLSTM branch classifier on the Yaseen dataset using 10-fold cross-validation.

Classes		Performance metrics				
		Accuracy	Sensitivity	Specificity	Precision	F1-score
	AS	95.20%±0.38%	81.00%±1.10%	98.75%±0.26%	94.26%±0.85%	87.09%±0.72%
	MR	94.30%±0.43%	82.50%±1.02%	97.25%±0.31%	88.38%±0.98%	85.33%±0.84%
	MS	95.30%±0.39%	94.50%±0.96%	95.50%±0.29%	84.33%±0.94%	89.02%±0.79%
	MVP	93.50%±0.41%	88.00%±1.08%	94.87%±0.27%	81.21%±0.93%	84.39%±0.77%
	Normal	98.70%±0.39%	96.50%±1.09%	99.25%±0.27%	97.16%±0.90%	96.75%±0.78%
**Average**		**95.40%±0.40%**	**88.50%±1.05%**	**97.12%±0.28%**	**89.07%±0.92%**	**88.51%±0.78%**

Bold values indicate the macro-average performance across all classes.

**Figure 16:**
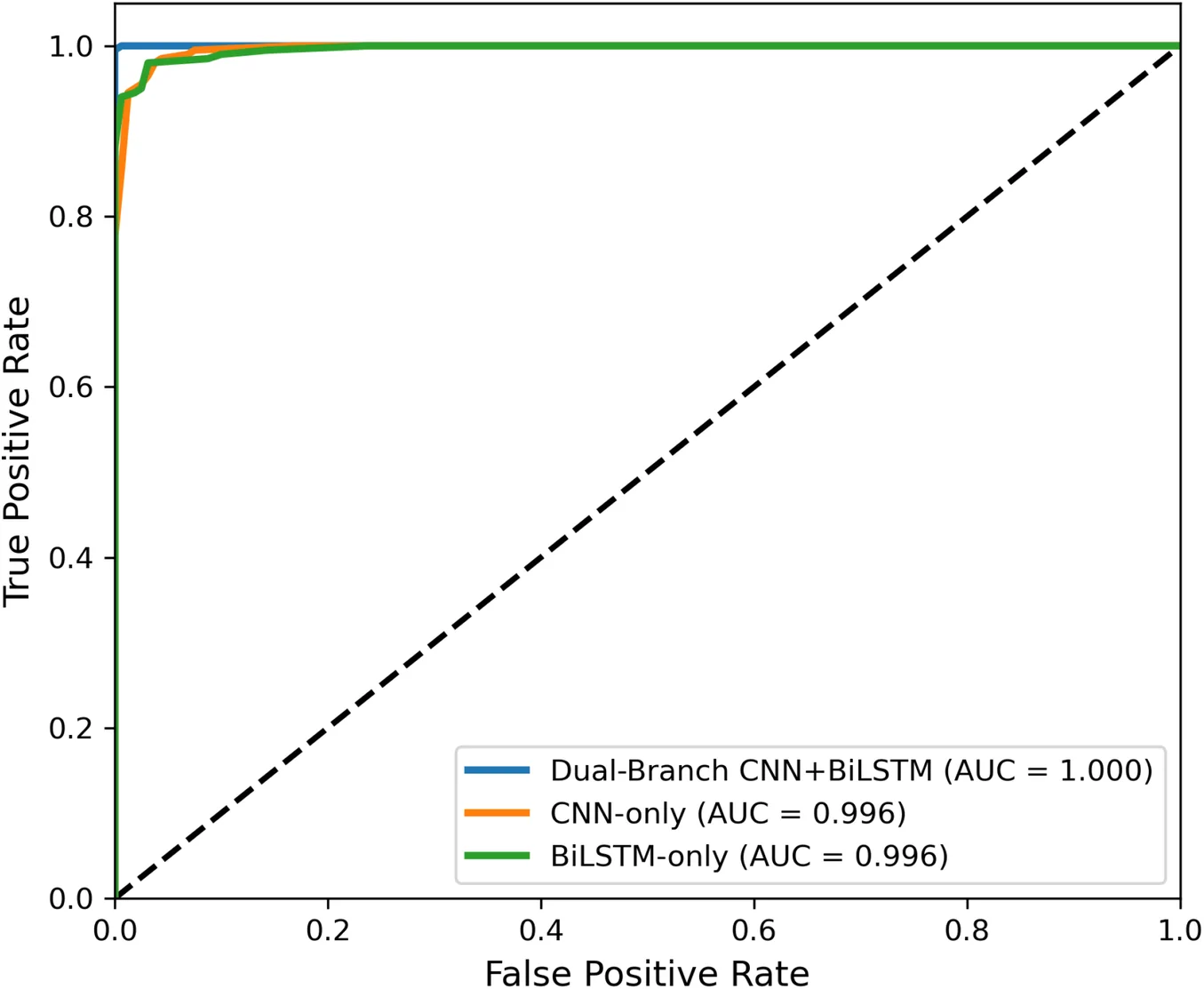
ROC curves and AUC scores of different models on the Yaseen dataset.

To further evaluate the threshold-independent discrimination ability of different models on the Yaseen dataset, we additionally plotted the ROC curves, as shown in Figure 16. Since Yaseen is a five-class classification task, ROC analysis was conducted in a one-vs.-rest manner, and the AUC values were macro-averaged across classes. The proposed HSD-Net achieved the highest AUC of 1.000, slightly outperforming both the CNN-only model (AUC = 0.996) and the BiLSTM-only model (AUC = 0.996). Overall, the ROC curves of all three models are concentrated near the upper-left corner, indicating strong discriminative ability on this dataset. This can be attributed, at least in part, to the relatively clean recordings in the Yaseen dataset, as well as the effectiveness of the adopted hybrid cepstral features in capturing class-discriminative acoustic characteristics. As a result, the differences among the curves are relatively small. Nevertheless, the ROC curve of HSD-Net remains consistently closer to the upper-left boundary, suggesting a more favorable trade-off between true positive rate and false positive rate across a wide range of decision thresholds. These findings further confirm that the proposed dual-branch fusion strategy provides stronger and more stable discriminative capability than either single-branch model alone.

Similarly, considering that the Yaseen dataset primarily contains low-noise recordings, we applied the same noise-injection protocol to the Yaseen dataset to further examine the robustness of HSD-Net on the secondary five-class benchmark. Noise samples from the MUSAN database (30) were added to the heart sound recordings at signal-to-noise ratio (SNR) levels of +20 dB, +10 dB, and +5 dB. This experiment was designed to assess whether the noise tolerance observed on the PhysioNet/CinC dataset could also be maintained under the multiclass classification setting. The corresponding results are summarized in Table 14. As the noise level increased, the classification performance gradually decreased, which is consistent with the results observed on the PhysioNet/CinC dataset. Nevertheless, HSD-Net still maintained relatively stable performance under the simulated noisy conditions. For example, in the +5 dB high-noise scenario, the model’s F1-score remained above 89.75%. These results further suggest that HSD-Net retains a certain degree of noise tolerance under controlled noise-injection conditions, while validation on real-world noisy clinical recordings remains necessary.

**Table 14:** Performance comparison of HSD-Net on the Yaseen dataset under clean and MUSAN-noise conditions at SNR levels of +20 dB, +10 dB, and +5 dB using 10-fold cross-validation.

SNR condition	Accuracy	Sensitivity	Specificity	Precision	F1-score
**Clean**	**99.32%±0.20%**	**98.30%±0.45%**	**99.57%±0.16%**	**98.34%±0.32%**	**98.30%±0.30%**
+20 dB	96.00%±0.48%	95.60%±0.50%	96.40%±0.45%	95.20%±0.55%	95.40%±0.52%
+10 dB	93.00%±0.75%	92.30%±0.80%	93.70%±0.70%	92.00%±0.85%	92.15%±0.83%
+5 dB	91.00%±0.95%	90.00%±1.00%	92.00%±0.90%	89.50%±1.05%	89.75%±1.02%

Bold values indicate the best performance.

### 3.8 Performance comparison with state-of-the-art methods

In this section, we compare the proposed algorithm with several state-of-the-art heart sound classification methods on two benchmark datasets. For related studies that reported results with multiple classifiers or feature sets, only the best-performing configuration was included for a fair comparison. The comparative results on the PhysioNet/CinC Challenge 2016 dataset and Yaseen dataset are summarized in Tables 15, 16, respectively.

**Table 15:** Classification performance comparison with existing methods on PhysioNet dataset.

Features	Method	Accuracy	Sensitivity	Specificity
PCG (33)	1D-CNN	86.80%	95.00%	86.00%
MFCCs (31)	CNN	91.50%	98.33%	84.67%
PCG (16)	CNN	86.80%	87.00%	72.10%
PCG (34)	CNN-BiLSTM	87.31%	92.78%	79.48%
Frequency (35)	SFTF and CNN	86.00%	88.70%	86.40%
Bispectrum (32)	CNN and Transformer	93.01%	74.72%	97.89%
WST (36)	CNN	96.10%	95.00%	95.00%
**Proposed features**	**HSD-Net**	**96.25%**	**95.85%**	**96.50%**

Bold values indicate the performance of the proposed method.

**Table 16:** Classification performance comparison with existing methods on Yaseen dataset.

Methods	Accuracy
Composite classifier (37)	98.33%
1D CNN (38)	98.60%
ConvNext (39)	98.70%
Spectrogram transformer (40)	98.74%
Vision transformer (41)	99.00%
Paramps (42)	99.20%
**Proposed HSD-Net**	**99.32%**

Bold values indicate the performance of the proposed method.

On the PhysioNet/CinC 2016 dataset, the proposed HSD-Net achieved the highest overall classification accuracy among all compared methods, while maintaining a favorable balance between sensitivity and specificity. It is worth noting, however, that some existing methods showed advantages in individual metrics. For example, Han et al. (31) reported higher sensitivity using an MFCC based method, whereas Wang et al. (32) reported higher specificity using a bispectrum based method. These differences can be explained from the perspective of the signal response characteristics of different feature representations. Specifically, the higher sensitivity of the MFCC based method is mainly related to its reliance on MFCC as the core feature representation. MFCC mainly characterize the low and middle frequency cepstral envelope of phonocardiogram signals, where the dominant components of the first and second heart sounds, as well as some abnormal acoustic variations, are usually concentrated. Therefore, a classifier mainly relying on MFCC may respond more strongly to spectral envelope variations and may be more likely to classify recordings as abnormal, thereby improving sensitivity. However, normal inter subject variability, changes in recording conditions, and residual noise may also cause envelope fluctuations, which can increase false positive decisions and reduce specificity. In contrast, although HSD-Net also uses MFCC, it does not treat them as the only dominant feature. Instead, it further fuses IMFCC and first order delta coefficients to introduce complementary high frequency features and short term dynamic information. Therefore, the MFCC based method can obtain higher sensitivity, whereas HSD-Net achieves a more balanced tradeoff between sensitivity and specificity and obtains higher overall accuracy.

For the bispectrum based method, its higher specificity is mainly related to the ability of bispectrum features to characterize higher order spectral correlations and phase coupling information, which helps identify relatively regular and stable acoustic structures in normal heart sounds. However, pathological murmurs are often transient, weak, and unevenly distributed in the time frequency domain, and may not necessarily form stable higher order coupling patterns. Therefore, bispectrum features may be less sensitive to some abnormal samples, resulting in lower sensitivity.

On the Yaseen dataset, HSD-Net also demonstrates strong performance across the five valvular heart sound categories. These results indicate that the proposed HSD-Net provides competitive and balanced classification performance, supporting its effectiveness for heart sound classification in screening-oriented applications.

## 4 Discussion

Based on the results of the above experiments, the following observations were drawn:

The approach presented in this study enables the use of automated heart sound classification for screening purposes. It provides technical guidance for developing efficient and affordable diagnostic tools based on automated heart sound analysis. In addition, it may serve as a foundation for further research in more complex clinical settings and multimodal cardiovascular disease.

Compared with existing single acoustic feature extraction methods, the hybrid cepstral fusion features achieve a more comprehensive characterization of heart sound signals. Specifically, traditional MFCC features primarily focus on spectral-envelope information in the mid-to-low frequency range and have been widely adopted in heart sound classification. However, many pathological murmurs manifest as faint high-frequency components, which may be relatively attenuated on the Mel-frequency scale. IMFCC effectively addresses this limitation. Furthermore, the differential features extracted from both methods capture short-term acoustic dynamics, ultimately leading to optimal classification performance.

The proposed deep learning framework, HSD-Net, aims to enhance classification accuracy for screening applications through feature fusion. CNN and BiLSTM play complementary roles in heart sound classification. Specifically, the CNN branch specializes in learning local and hierarchical structural patterns from 2D time-frequency feature maps, such as distinctive differences in spectral texture between normal and abnormal heart sounds. The BiLSTM branch, through its bidirectional recurrent structure, captures temporal dependencies over prolonged time intervals, enabling modeling of multiple cardiac cycles and the temporal relationships among pathological events. Ablation experiments confirm this complementarity: under identical training conditions, models relying solely on the CNN branch or solely on the BiLSTM branch demonstrate inferior screening performance compared with the complete dual-branch architecture. Furthermore, integrating the SE attention module at both the convolutional and fusion stages highlights channels that are vital for differentiating normal vs. abnormal heart sounds or distinct valvular pathologies, thereby yielding further improvements in screening accuracy.

The experimental results on two public datasets demonstrated the effectiveness of HSD-Net in heart sound classification tasks designed for clinical screening. On the PhysioNet/CinC Challenge 2016 dataset, the proposed method achieved competitive sensitivity, specificity, and accuracy compared with multiple traditional machine learning and deep learning baselines. On the Yaseen dataset, the model also achieved strong performance in the five-class valvular heart disease classification task. These results collectively support the effectiveness of HSD-Net for automated heart sound screening.

### 4.1 Limitations and future work

Although cross-validation was employed to enhance the reliability of performance estimation, several limitations remain. First, the overall scale of the datasets used in this study is still relatively limited, which may have a potential influence on the generalization ability of the proposed model. In heart sound classification, the influence of dataset size is reflected not only in the absolute number of recordings, but also in whether the available data sufficiently cover the physiological, pathological, device related, and environmental variability of PCG signals. Limited data diversity may make it difficult for the training and testing process to fully represent different patient populations, disease subtypes, comorbidity profiles, heart rate patterns, recording devices, acquisition protocols, and environmental noise conditions. Although the PhysioNet/CinC Challenge 2016 dataset includes recordings collected from multiple sources and the Yaseen dataset provides an additional five class benchmark, these public datasets may still have limited coverage of rare pathological acoustic patterns, borderline cases, device specific signal characteristics, and low quality recordings that may occur in broader clinical screening scenarios. Such limited coverage may influence model generalization in several aspects. First, the model may have fewer opportunities to learn stable acoustic representations that are transferable across different populations, devices, and acquisition environments. Second, the learned decision boundaries may be more dependent on the distribution of the evaluated datasets; therefore, when external data differ in disease composition, signal quality, or noise characteristics, the balance between sensitivity and specificity may be affected. Third, under potential distribution shifts, the reliability of model confidence for atypical cases and low quality recordings still requires further assessment. In addition, while the fixed length segmentation strategy is straightforward and efficient, it may not fully accommodate inter subject variability in heart rate and cardiac cycle duration, and may occasionally truncate physiologically informative acoustic events. Class weights were adopted to alleviate class imbalance during training, but the effects of different imbalance handling strategies, class weighting schemes, and decision thresholds were not systematically compared. In addition, some feature design parameters, such as the number of Mel filters and retained cepstral coefficients, were mainly determined according to common practice and empirical settings, and therefore require further sensitivity analysis.

To address these limitations, future research will focus on several directions. First, HSD-Net should be further evaluated on larger scale, multi center, and prospectively collected clinical PCG datasets to more comprehensively assess its generalization ability across diverse patient populations, disease subtypes, acquisition devices, recording protocols, and signal quality conditions. Particular attention should be paid to the inclusion of rare pathological acoustic patterns, borderline cases, device specific signal characteristics, and low quality recordings that may occur in real world clinical screening. More recent PCG datasets, such as the PhysioNet/CinC Challenge 2022 dataset, may also be considered after appropriate task adaptation, since its murmur detection setting differs from the classification tasks investigated in this study. Second, physiologically aligned segmentation strategies should be explored to better handle heart rate variability and reduce the risk of discarding clinically relevant acoustic events. Finally, future studies should systematically assess the effects of class imbalance handling methods, decision threshold selection, feature extraction parameters, and key model components to further improve the methodological rigor of the proposed framework.

### 4.2 Ethical considerations and clinical risk analysis

Although this study uses publicly available datasets, deploying AI-based heart sound analysis for real-world cardiovascular screening still raises important ethical and clinical safety considerations. In particular, false negatives may lead to more severe consequences: incorrectly classifying individuals with potential cardiac abnormalities as normal can delay further evaluation or referral, potentially missing early intervention windows and increasing the risk of adverse outcomes. Therefore, the proposed method should be positioned as a screening/triage aid rather than a standalone diagnostic tool, and deployment should prioritize strategies that reduce missed cases, such as selecting a sensitivity-oriented decision threshold for screening, flagging low-confidence or poor-quality recordings for re-examination instead of returning a negative result, and maintaining clinician review in conjunction with history and physical findings. Future work will further quantify false-negative risk through prospective evaluation and deployment-oriented testing to improve clinical safety and reliability.

## 5 Conclusions

In clinical practice, patients with latent cardiac diseases are often overlooked because their symptoms are subtle, leading to delays in optimal diagnosis and treatment. This not only allows the disease to progress but also increases the complexity and cost of subsequent interventions. To enable accurate and efficient early screening of cardiac diseases, this study proposes HSD-Net, an automated heart sound screening framework that integrates hybrid cepstral fusion features with a CNN-BiLSTM deep neural network. The proposed method was systematically evaluated on two public datasets. On the PhysioNet/CinC Challenge 2016 dataset, it achieved an accuracy of 96.25%, with sensitivity and specificity of 95.85% and 96.50%, respectively. On the Yaseen dataset, the average accuracy across five types of valvular heart disease reached 99.32%, with sensitivity and specificity of 98.30% and 99.57%. The F1-scores for all categories remained consistently high, indicating that the method shows stable benchmark performance across the evaluated task settings. In practical applications, the heart sound screening results generated by HSD-Net can provide valuable supplementary information for clinicians. In particular, among individuals without obvious subjective symptoms but at risk of underlying cardiac pathology, model outputs indicating abnormal or suspicious heart sounds can serve as early warning signals that warrant closer clinical attention and timely referral for further diagnostic workup, such as echocardiography or other advanced cardiac imaging. In this way, detection rates and opportunities for early intervention in high-risk populations can be improved without substantially increasing healthcare workload or cost. Especially in primary care settings and resource-limited regions, automated analysis systems based on digital auscultation and deep learning can serve as important adjunct tools for cardiovascular disease screening, follow-up monitoring, and telemedicine applications.

## Data Availability

Publicly available datasets were analyzed in this study. This data can be found here: the datasets analyzed in this study are publicly available. The PhysioNet/CinC Challenge 2016 dataset is available at https://physionet.org/content/challenge-2016/1.0.0/. The Yaseen heart sound dataset is available at https://github.com/yaseen21khan/Classification-of-Heart-Sound-Signal-Using-Multiple-Features-/blob/master/README.md.
